# Elucidating the Role of MicroRNA-18a in Propelling a Hybrid Epithelial–Mesenchymal Phenotype and Driving Malignant Progression in ER-Negative Breast Cancer

**DOI:** 10.3390/cells13100821

**Published:** 2024-05-10

**Authors:** Madhumathy G. Nair, Apoorva D. Mavatkar, Chandrakala M. Naidu, Snijesh V. P., Anupama C. E., Savitha Rajarajan, Sarthak Sahoo, Gayathri Mohan, Vishnu Sunil Jaikumar, Rakesh S. Ramesh, Srinath B. S., Mohit Kumar Jolly, Tessy Thomas Maliekal, Jyothi S. Prabhu

**Affiliations:** 1 Division of Molecular Medicine, St. John’s Research Institute, St. John’s Medical College, Bangalore 560034, Karnataka, India; 2 Department of Bioengineering, Indian Institute of Science (Bangalore), Bengaluru 560012, Karnataka, India; 3 Cancer Research, Rajiv Gandhi Centre for Biotechnology (RGCB), Thiruvananthapuram 695014, Kerala, India; 4 Animal Research Facility, Rajiv Gandhi Centre for Biotechnology (RGCB), Thiruvananthapuram 695014, Kerala, India; 5 Department of Surgical Oncology, St. John’s Medical College and Hospital, Bangalore 560034, Karnataka, India; 6 Department of Surgical Oncology, Sri Shankara Cancer Hospital and Research Centre, Bangalore 560004, Karnataka, India

**Keywords:** microRNA-18a, epithelial–mesenchymal transition, ER-negative breast cancer, hybrid E/M phenotype, chemoresistance, stem-like cells

## Abstract

Epigenetic alterations that lead to differential expression of microRNAs (miRNAs/miR) are known to regulate tumour cell states, epithelial–mesenchymal transition (EMT) and the progression to metastasis in breast cancer. This study explores the key contribution of miRNA-18a in mediating a hybrid E/M cell state that is pivotal to the malignant transformation and tumour progression in the aggressive ER-negative subtype of breast cancer. The expression status and associated effects of miR-18a were evaluated in patient-derived breast tumour samples in combination with gene expression data from public datasets, and further validated in in vitro and in vivo breast cancer model systems. The clinical relevance of the study findings was corroborated against human breast tumour specimens (n = 446 patients). The down-regulated expression of miR-18a observed in ER-negative tumours was found to drive the enrichment of hybrid epithelial/mesenchymal (E/M) cells with luminal attributes, enhanced traits of migration, stemness, drug-resistance and immunosuppression. Further analysis of the miR-18a targets highlighted possible hypoxia-inducible factor 1-alpha (HIF-1α)-mediated signalling in these tumours. This is a foremost report that validates the dual role of miR-18a in breast cancer that is subtype-specific based on hormone receptor expression. The study also features a novel association of low miR-18a levels and subsequent enrichment of hybrid E/M cells, increased migration and stemness in a subgroup of ER-negative tumours that may be attributed to HIF-1α mediated signalling. The results highlight the possibility of stratifying the ER-negative disease into clinically relevant groups by analysing miRNA signatures.

## 1. Background

Estrogen Receptor (ER) is an important regulator of mammary growth and development. Loss of ER function has been linked to the emergence of endocrine resistance and poor prognosis in the ER-positive breast cancer [BC] subtype [1,2]. ER-negative breast cancer is featured by the absence of ER expression and is associated with a poor prognosis, aggressive disease, and early relapse in comparison to the ER-positive subtype [3]. Despite an initial response to chemotherapy, which is the main modality of treatment for the ER-negative subtype, there is a high risk of recurrence and distant metastasis [4]. The ER-negative subtype is also heterogeneous at the pathological, clinical and at the molecular level with a mutational profile vastly distinct from other subtypes [5,6]. Epigenetic alterations resulting in deviant gene expression profiles are also key contributors to the process of tumour progression in ER-negative breast cancer [7].

Deregulated expression of small non-coding regulatory RNA molecules known as microRNAs has been attributed to cause epigenetic alterations that can affect the process of tumour progression [8]. miRNAs control the process of gene expression and are implicated in the process of aggressive disease progression in ER-negative as well as in the triple negative subtype of breast cancer [9,10]. miRNAs like miR-34 and miR-200 have also been linked to the regulation of plasticity required for cells to transition between the various phases of epithelial to mesenchymal transition (EMT) [11]. EMT drives tumour cells to undergo transformations critical for migration, immune evasion, distant organ seeding and eventually metastasis [12]. This plasticity enables the cells to shuttle between epithelial, mesenchymal and the epithelial–mesenchymal hybrid (E/M hybrid) phenotypes and is termed epithelial–mesenchymal plasticity (EMP). The E/M hybrid state consists of cells that are highly tumorigenic and stem cell-like. In addition, they also possess features of both epithelial and mesenchymal cells that enable collective cell migration, immune evasion and higher tumour-initiating ability which are factors associated with poor clinical prognosis [13].

miR-18a belongs to the miR-17-92 cluster and has been reported to play a key role in the malignant progression of multiple cancer types including lung, gastric, cervical, prostate, breast cancer and osteosarcoma [14]. miR-18a is also reported to have a multifaceted role in tumour progression. It has been reported to promote cancer progression in non-small-cell lung cancer, cervical cancer and prostate cancer. On the contrary, it has shown to play tumour-suppressor roles in pancreatic and colorectal cancer [15]. We have previously demonstrated that high levels of miR-18a promoted poor prognosis in ER-positive breast cancer by activating Wnt signalling and bringing about actin remodelling and immune suppression [16,17]. The high levels of miR-18a expression and its effect on prognosis and drug resistance in triple negative breast cancers has been reported previously [18,19]. Here, we report for the first time, a dual functional role of miR-18a in breast cancer that is subtype specific and dependent on the expression status of hormone receptors. The study also sheds light on the novel association of low miR-18a levels and the enrichment of hybrid E/M cells that leads to phenotypic changes including that of increased migration and stemness in a subgroup of ER-negative tumours.

## 2. Materials and Methods

### 2.1. Cell Lines, Culture and Transfection with miR-18a Synthetic Inhibitors

The cell line MDA-MB-468 was obtained from the National Centre for Cell Science (Pune, Maharashtra, India) where cell authentication was performed using short tandem repeat profiling. MDA-MB-468 was cultured in Dulbecco’s Modified Eagle Medium (DMEM) (Sigma-Aldrich, St. Louis, MO, USA) supplemented with 10% Foetal Bovine Serum (FBS) (Himedia, Thane, Maharashtra, India). MDA-MB-231 was obtained from the American Type Culture Collection (ATCC, Manassas, VA, USA). MDA-MB-453 (an ER-negative Her2 positive cell line) was obtained as a gift from Dr. Annapoorni Rangarajan, (Indian Institute of Science, Bengaluru, Karnataka, India). For all experimental assays using cell lines, a passage number below 20 was used and all cell lines were subjected to frequent recharacterization by immunophenotyping and testing of mycoplasma.

microOFF™ miRNA inhibitor for miR-18a was purchased from Guangzhou RiboBio Co., Ltd. (Science City, Guangzhou, China). hsa-miR-18a-5p antagomiR was purchased from Shanghai GenePharma Co., Ltd. (Pudong New Area, Shanghai, China). The miR-inhibitor/antagomiR was transfected into cultured MDA-MB-468, MDA-MB-231 and MDA-MB-453 using Lipofectamine RNAiMAX Transfection Reagent (Invitrogen, Waltham, MA, USA) according to the manufacturer’s protocol. Briefly, 1 × 10^5^ cells were seeded in a 12-well plate in antibiotic free media with 10% FBS. The following day, microOFF™ miRNA inhibitor/hsa-miR-18a-5p antagomiR (hsa-miR-18a-5p CUAUCUGCACUA GAUGCACCUUA) were mixed with riboFECT™ CP Buffer. A nonspecific microOFF™ inhibitor negative control (cel-miR-239b-5p MIMAT0000295 UUUGUACUACACAAAAGUACUG) or antagomiR negative control was used as the scrambled or negative control. The final concentration of the inhibitor/antagomiR and scrambled was 50–100 nM. To this complex, 3 µL Lipofectamine RNAiMAX (Invitrogen, USA) was added and incubated for 45 min at room temperature (RT). The transfection complex was then added to the cells along with antibiotic free media with 10% FBS and full distribution over the plate surface was ensured. The cells were incubated for a period of 48–72 hours (h) before harvesting.

The cells after miR-18a inhibition will be referred to hereafter as MDA-MB-468/miR-18a/inh, MDA-MB-231/miR-18a/inh and MDA-MB-453/miR-18a/inh and the cells transfected with the negative control will be referred to as MDA-MB-468/miR-18a/cont, MDA-MB-231/miR-18a/cont and MDA-MB-453/miR-18a/cont. The transfection efficiency was evaluated by assessing the levels of the microRNA targets by Western blot and q-PCR after 48–72 h. For HIF-1α pathway inhibition, MDA-MB-468/miR-18a/inh cells were treated with a small molecule inhibitor of HIF-1α; CAY10585 (Abcam, Cambridge, UK), 4 h after transfection. After 72 h, cells were harvested for various assays.

### 2.2. Protein Expression Analysis by Western Blot

Post-transfection, the cellular protein expression was evaluated and densitometric analysis was performed using quantity one software (Magellan 7.1 sp1-Bio-Rad, Hercules, CA, USA) as reported previously [20]. The list of antibodies used are listed in the Supplementary Materials (Supplementary Table S1).

### 2.3. Immunophenotyping by Flow-Cytometry

Post-transfection, cells were trypsinised and, post-recovery, washed with PBS, fixed in 4% PFA for 10 min followed by permeabilisation in 0.2% Triton X-100 in PBS. Cells were then incubated at RT for 1 h in primary antibodies for CD44 and CD24 at specific dilutions (Supplementary Table S1) and then labelled with specific secondary antibodies. Cells were then re-suspended in 600 μL of PBS and analysed using a FACSCalibur cytometer (BD Biosciences, Franklin Lakes, NJ, USA). The percentage of CD44^high^ CD24^low^, CD44^low^ CD24^high^ and CD44^high^ CD24^high^ expressing cells were analysed. Appropriate secondary antibody controls were included for the analysis. The FL1-H channel was used to detect CD44 and the FL2-H channel was used for the detection of CD24.

### 2.4. Dual Immunofluorescence

Cells were seeded on poly-L-lysine-coated coverslips and transfected as described above. Immunofluorescence was performed as reported previously [20] by incubating cells in primary antibodies anti-E-cadherin and anti-Vimentin overnight at 4 °C at specific dilutions (Supplementary Table S1). This was followed by labelling with specific secondary antibodies—Alexa Fluor^®^ 488 Chicken Anti-Mouse IgG (H+L) for Anti-Vimentin and Alexa Fluor 568 Donkey Anti-Rabbit IgG for anti-E-cadherin for 1 h at RT. The slide was then mounted on gold antifade reagent with DAPI and examined under a fluorescent microscope (Olympus BX51, Shinjuku, Tokyo, Japan).

### 2.5. Computational Analysis for Correlation with E/M Hybrid Score

The ER-negative tumours of the TCGA-PanCancer Atlas (n = 211) and the METABRIC Nature 2012 and Nat Commun 2016 cohorts (n = 265) were segregated based on the upper and lower quartiles of miR-18a expression. The TCGA series with n = 50 (miR-18a/low) and n = 57 (miR-18a/high) tumours and the METABRIC series with n = 54 (miR-18a/low) and n = 62 (miR-18a/high) tumours were used for further analysis. The clinico-pathological features of the tumours used for the study are enlisted in Supplementary Tables S2 and S3. The TCGA data were accessed from the TCGA Research Network: https://www.cancer.gov/tcga (accessed on 15 November 2020), and the METABRIC data were accessed from the European Genome-phenome Archive [21]. We used four gene signatures to score the individual patient samples to characterize their luminal, basal, epithelial and mesenchymal characteristics. The gene lists for the luminal and basal signature were obtained from a cumulative list of genes (Supplementary Table S4) listed from previously published reports [22,23,24,25,26] and the gene lists for the epithelial and mesenchymal programs were obtained from Tan et al., EMBO Mol. Med. 2014 [27]. To calculate the scores, we used the ssGSEA algorithm [28] present as a part of the gseapy Python package(1.0.5).

### 2.6. Breast Tumour Specimens Used for Gene Expression Analysis

Tumour samples used for molecular analysis were obtained from surgically excised breast tumour specimens from 446 patients enrolled prospectively at two tertiary-care hospitals (St. John’s Medical College and Hospital and Rangadore Memorial Hospital) in Bangalore, from June 2008 to February 2013. Informed consent for use of the material for research was obtained from all patients and the study was approved by the IERB (Institutional Ethics Review Board) at both hospitals (St. John’s Medical College and Hospital (No. 62/2008) and Rangadore Memorial Hospital (RMHEC/02/2010)). Samples were fixed in 10% neutral buffered formalin at RT and stored as formalin-fixed paraffin-embedded (FFPE) blocks. From the set of treatment, naive tumour samples (n = 275), ER-negative tumour blocks (n = 105) and ER-positive tumour blocks (n = 170) that met quality control (QC) criteria for molecular analysis were used for mRNA and miRNA expression analysis. The clinico-pathological features of the ER-negative tumours used for the study are enlisted in Supplementary Table S5.

A set of tissue samples from surgically excised breast tumours with residual disease post-neoadjuvant chemotherapy (NACT) including partial and non-responders (n = 54) have also been used. All patients were treated with chemotherapy regimens that included anthracyclines and/or taxanes. Of the 54 residual sections, 43 had adequate tissue for further analysis. Of the 43, 24 qualified for miRNA expression analysis and 34 had sufficient tissue for performing IHC. The clinico-pathological features of the tumours used for the study are enlisted in Supplementary Table S6.

### 2.7. mRNA and miRNA Expression Analysis Using Quantitative PCR

Extraction of RNA, cDNA synthesis and q-PCR experiments were performed on tumour specimens and cell line lysates as reported previously [20]. The primer sequences for the genes tested are given in Supplementary Table S7. miRNA present in total RNA was extracted and converted to cDNA using stem-loop primers specific for the chosen miRNA as described previously [16]. miRNA-U48 was used as an endogenous control for normalisation.

### 2.8. Analysis of Mutational Spectrum of Breast Tumours of the METABRIC Cohort

The ER-negative tumours of the METABRIC Nature 2012 and Nat Commun 2016 cohorts (n = 265) were segregated based on the upper and lower quartiles of miR-18a expression into n = 54 (miR-18a/low) and n = 62 (miR-18a/high) tumours. The mutational spectrum of cancer driver genes which were collected from the IntoGen database was examined [29]. The deleterious variants with IMPACT ‘HIGH’ or ‘MODERATE’ were only considered for the analysis. The genes were selected if mutated at least three times across samples. Fisher’s exact test was performed to confirm the significance of the mutations between the miR-18a/low and high tumour samples.

### 2.9. Analysis of Differentially Expressed Genes (DEGs) and Pathways in Breast Tumours of the TCGA and METABRIC Series

The ER-negative tumours of the TCGA-PanCancer Atlas (n = 211) and the METABRIC Nature 2012 and Nat Commun 2016 cohorts (n = 265) were segregated based on the upper and lower quartiles of miR-18a expression as described above. Significant DEGs between miR-18a/high and miR-18a/low groups were filtered based on absolute fold change (FC) ≥ 2 and adjusted *p* ≤ 0.05. Gene ontology and pathway analysis of DEGs were performed using the ToppGene suite [30]. The deregulated pathways derived from DEGs were visualised using GOplot-r packages (1.0.2) [31].

### 2.10. Correlative Analysis of Published EMT Scores with miR-18a Expression in Breast Tumours of the TCGA and METABRIC Series

The TCGA series with n = 50 (miR-18a/low) and n = 57 (miR-18a/high) tumours and the METABRIC series with n = 54 (miR-18a/low) and n = 62 (miR-18a/high) tumours were used for this analysis. A pan-cancer EMT signature derived from the patient–tumour data of 11 different cancer types [32] was used for analysing the association with miR-18a. Additionally, a core-gene list of 130 EMT-related genes derived from a meta-analysis of 10 GES datasets was also used for this analysis [33].

### 2.11. In Vitro Cell Migration—Wound Closure Assay

Cells were transfected as described above. Forty-eight hours after transfection of cells, the media were replaced with low serum media (0.2% Foetal Bovine Serum) and cells were allowed to rest for 6 h. A wound was generated, and images were captured to mark the initiation time (0 h) and after 48 h. The migratory ability was quantified and normalized by measuring the relative gap distance and compared between cells transfected with microOFF™ miRNA inhibitor and negative control.

### 2.12. Immunohistochemistry of Residual Tumours to Evaluate Expression of Integrin β3

Tissue samples from surgically excised breast tumours with residual disease post-neoadjuvant chemotherapy (NACT) including partial and non-responders (n = 34) were used for immunohistochemistry. The primary antibody for integrin β3 was applied for 1 h at RT. Sections were further incubated with the secondary antibody (DAKO REALTM EnVisionTM, Glostrup, Denmark) for 20 min at RT as per the kit instructions, followed by development of the colour using DAB (DAKO REALTM EnVisionTM) for 10 min. Appropriate positive and negative controls were run for each batch. Staining patterns of integrin β3 were evaluated by a pathologist (J.S.P). The protein expression analysis was performed on post-NACT specimens of patients who had a partial response to chemotherapy where the tumour specimens have more stromal component and less tumour. Hence, immunoreactivity of more than 1% of the residual tumour epithelial cells was considered as positive expression for integrin β3.

### 2.13. Evaluation of Drug Cytotoxicity Using MTT

MDA-MB-468 cells were seeded (2 × 10^4^) in 96-well microtiter plates and transfected as described above. After 48 h, the medium was removed and replaced with 100 μL of media with paclitaxel at various doses from 10 μM to 200 μM for 48 h.

MDA-MB-231 cells were transfected with ALDH1A1-DsRed2N1 plasmid using Lipofectamine2000 as described previously [34]. The stably transfected cells were selected with 100 µg/mL Geneticin (G418) and sorted out in FACS Aria II to enrich CSCs, which were then maintained (20 µg/mL of G418) for experimental purpose. These cells were obtained as a gift from T.T.M. miR-18a was inhibited in these cells and the control (DsRed2N1) cells using microOFF™ inhibitor and inhibitor negative control as described above. After 72 h, the medium was removed and replaced with 100 μL of media with paclitaxel at various doses from 10 μM to 200 μM for 48 h. MTT assay was performed as reported previously [20]. The selectivity index (SI) has been calculated according to a previously reported publication [35].

### 2.14. Generation of Mammospheres and Extreme Limiting Dilution Assay (ELDA) to Assess Clonogenicity

Transfection was performed as described above and 72 h post-transfection, MDA-MB-468/miR-18a/inh, MDA-MB-453/miR-18a/inh, MDA-MB-468/miR-18a/cont and MDA-MB-453/miR-18a/cont cells were trypsinised and seeded to form spheres using DMEM/F12 media supplemented with 20 ng/mL FGF and EGF along with insulin–transferrin supplement in low adherent 12-well plates coated with Poly (2-hydroxyethyl methacrylate). After 5 days, the first-generation spheres were serially propagated and reseeded to form second-generation spheres in low adherent 96-well plates by serial dilution. Cells were seeded at a frequency of 1000, 500, 100, 10 and up to 1 cell/well in sextuplicate. After 6 days, the spheres were counted and the sphere-forming ability was calculated using the extreme limiting dilution analysis (ELDA) algorithm as previously described [36].

### 2.15. Estimate Analysis and Immune Cell Identification

The ER-negative tumours of the TCGA-PanCancer Atlas (n = 211) and the METABRIC Nature 2012 and Nat Commun 2016 cohorts (n = 265) were segregated based on the upper and lower quartiles of miR-18a expression. The TCGA gene expression data were used to infer the stromal and immune scores to predict the level of infiltrating stromal and immune cells in the tumours along with the cumulative ESTIMATE score using the ESTIMATE algorithm [37]. The normalized gene expression data with standard annotation files from the TCGA and the METABRIC cohorts were also used for the deconvolution of infiltrating immune populations by the CIBERSORT algorithm as described previously [17]. CIBERSORT was run with the following options: relative and absolute modes together, LM22 signature gene file, 1000 permutations and quantile normalization disabled. Using the filtered data, the proportions of immune cells in the miR-18a/high and miR-18a/low breast tumours were displayed in the form of a proportion plot. The normalized gene expression data with standard annotation files from the TCGA and the METABRIC cohort were also uploaded to the Immune Cell Abundance Identifier (ImmuCellAI), to precisely estimate the infiltration score of 24 immune cell types, including 18 T-cell subsets [38].

### 2.16. Breast Xenograft in In Vivo Studies

miR-18a was inhibited in MDA-MB-468 using hsa-miR-18a-5p antagomiR and antagomiR negative control as described above. After 72 h, 0.5 × 10^6^ cells from each were harvested and suspended in 50 μL of PBS. Mice were randomly distributed into antagomiR (n = 10) and control (n = 10) groups. Orthotopic tumours were induced by exposing the fourth (inguinal) mammary fat pad of female NSG/NOD-SCID mice at 6–7 weeks of age (bred and maintained at Rajiv Gandhi Centre for Biotechnology, Thiruvananthapuram, Kerala, India (326/GO/ReBiBt/S/2001/CPCSEA) and injecting them with cells suspended in 50 μL of Matrigel. On observing palpable tumours, the mice were sacrificed after 21 days, and tumour samples were harvested, and weight measured.

### 2.17. Histopathological Analysis and Immunostaining of Mice-Derived Tumours

Mouse tumours obtained as described above (n = 5 from each group) were formalin-fixed, paraffin-embedded, 5 µm sections were cut and haematoxylin/eosin staining was performed following the standard protocol. Tumours (n = 5 from each group) were also fixed in 4% PFA and mounted in OCT compound. Sections of 8 μm were taken and permeabilized with 0.3% Triton X-100 in PBS and blocked with 3% serum. Primary antibody was added at specific dilutions (Supplementary Table S1) and immunofluorescence performed with anti-E-cadherin and anti-Vimentin antibodies as described above.

### 2.18. miR-18a Target Prediction

We analysed the potential targets of miR-18a with six different databases and miRNA target prediction tools—miRanda, TargetScan, microT-CDS, PicTar, miRTarBase and miRDB. The common miR-18a targets predicted by at least three prediction programs were selected for further analysis. The mRNA levels of these targets and their association with miR-18a transcript levels were examined in the miR-18a/low and high samples of the TCGA and the METABRIC series.

### 2.19. Statistical Analysis

Descriptive statistics were used for all clinical variables. The difference in gene expression levels was evaluated by the Mann–Whitney U test/Kruskal–Wallis test or the two-tailed Student’s *t*-test. Correlations were evaluated by Pearsons’ rank test. Kaplan–Meier analysis was used to examine the estimated differences in disease-free survival between the miR-18a/high and miR-18a/low groups. Log-rank test (Mantel–Cox) was used to compare the survival between groups. For in vitro experimentations, the results are depicted as mean ± standard error of the mean calculated from three independent experiments and statistical analysis was performed using Student’s *t*-test. For all tests, *p* < 0.05 was considered to be statistically significant. All statistical analysis was carried out using the software XLSTAT 2022.2.1.

## 3. Results

### 3.1. Low Levels of miR-18a Enriches for the Hybrid Epithelial/Mesenchymal–Lumino/Basal Phenotype in ER-Negative Breast Cancer

**Figure 1:**
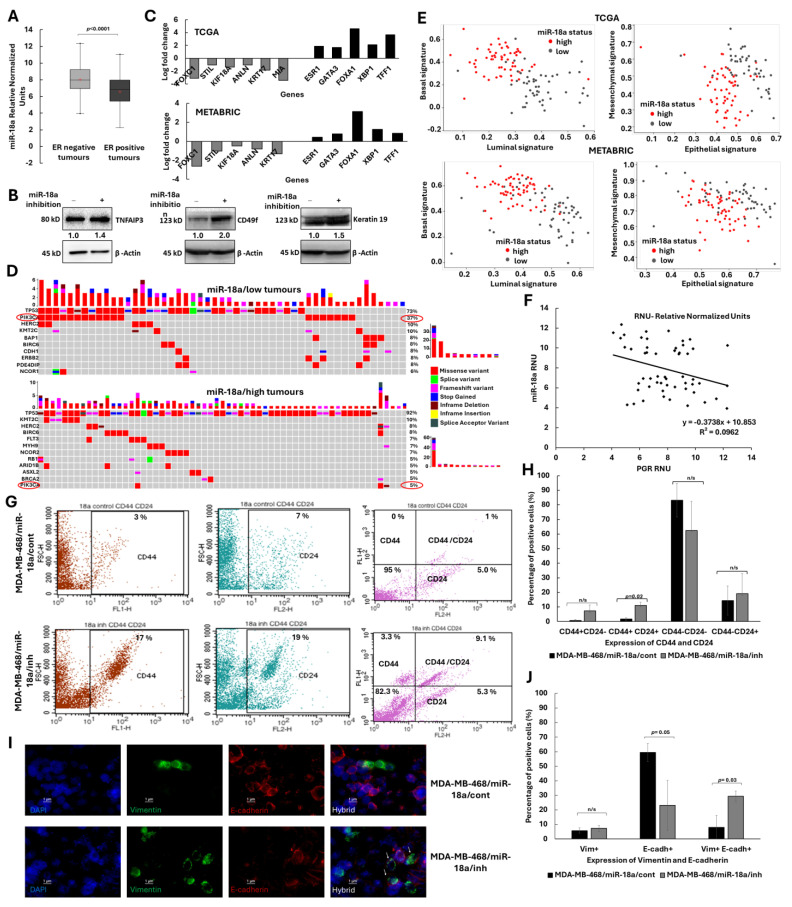
Reduced levels of miR-18a enriches for hybrid EMT/luminal-basal phenotype in ER-negative breast cancer. (**A**) Expression levels of miR-18a transcripts in ER-negative (n = 105) and ER-positive (n = 170) tumours (part of our cohort) as examined by q-PCR. (**B**) Change in protein expression of TNFAIP3 between MDA-MB-468/miR-18a/cont and MDA-MB-468/miR-18a/inh and CD49f, Keratin 19 between MDA-MB-231/miR-18a/cont and MDA-MB-231/miR-18a/inh cells. (**C**) Expression levels of luminality- and basality-associated genes in miR-18a/low tumours of the TCGA and METABRIC cohorts when compared to miR-18a/high tumours in the ER-negative subtype. (**D**) Mutational spectrum analysis depicting higher *PIK3CA* mutation load in miR-18a/low in comparison to miR-18a/high ER-negative tumours of METABRIC cohort. (**E**) Gene signature-based computational analysis characterising luminal, basal, epithelial and mesenchymal scores/signatures in miR-18a/low and miR-18a/high, ER-negative tumours of the TCGA and METABRIC cohorts. (**F**) Correlation analysis between miR-18a and *PGR* transcript levels in the miR-18a/high (n = 24) and miR-18a/low (n = 30) ER-negative tumours (part of our cohort) as examined by q-PCR. (**G**) Representative immunophenotyping images depicting the expression levels of CD44 and CD24 in MDA-MB-468/miR-18a/cont and MDA-MB-468/miR-18a/inh cells as assessed by flow cytometry. (**H**) Quantitative assessment of CD44 and CD24 expression in MDA-MB-468/miR-18a/cont and MDA-MB-468/miR-18a/inh as assessed by flow cytometry (cumulative result from three independent trials). (**I**) Representative immunofluorescence images demonstrating the expression of E-cadherin and Vimentin in MDA-MB-468/miR-18a/cont and MDA-MB-468/miR-18a/inh. Arrows represent hybrid epithelial/mesenchymal cells. (**J**) Quantitative assessment of E-cadherin and Vimentin expression in MDA-MB-468/miR-18a/cont and MDA-MB-468/miR-18a/inh.

Evaluation of the levels of miR-18a in 275 breast tumour samples by q-PCR showed that miR-18a was highly expressed (*p* < 0.0001) in the ER-negative tumours (n = 105) when compared to ER-positive tumours (n = 170) (Figure 1A). The ER-negative tumours considered for analysis comprised of tumours featured by the absence of ER expression and presence/absence of the HER2 growth factor. To further probe the role of miR-18a in ER-negative tumours, miR-18a was inhibited using microOFF™ miRNA inhibitor in breast cancer cell lines. We measured the protein levels of TNFAIP3, an experimentally validated target of miR-18a to assess transfection efficiency. We have previously shown the effective repression of TNFAIP3 protein with miR-18a over-expression [16]. In MDA-MB-468/miR-18a/inh cells, we observed a 45% increase in the levels of TNFAIP3 (*p* = 0.0002, Figure 1B). The levels of other targets of miR-18a were assessed by q-PCR after miR-18a inhibition in both MDA-MB-468 and MDA-MB-231. The levels of miR-18a target genes *BIRC3*, *HIF1A*, *DICER* and *CDK19* increased in the cell lines after miR-18a inhibition (Supplementary Figure S1).

Since miR-18a is involved in epigenetic regulation of the estrogen receptor, we probed for the expression of Keratin 19 that is typically expressed in luminal epithelial cells. On miR-18a inhibition, Keratin 19 levels increased by 50% (*p* = 0.01) in MDA-MB-231 (Figure 1B). This increase in the levels of a luminal cytokeratin in an ER-negative cell line was intriguing and to examine the possibility of enrichment of luminal–basal hybrid cells, we analysed the ER-negative tumours of the TCGA and the METABRIC cohorts. The miR-18a/low tumours had higher expression of the luminality-associated genes like *ESR1*, *GATA3*, *FOXA1*, *XBP1* and *TFF1* and a lower expression of the basality-associated genes such as *KRT18*, *KRT17*, *FOXC1*, *ANLN*, *STIL* and *MIA* (*p* < 0.05) (Figure 1C). These tumours were also analysed for the mutational spectrum of the cancer driver genes and this analysis showed a significant mutation load of *PIK3CA* in mir-18a/low tumours when compared to mir-18a/high tumours (*p* = 2.15 × 10^−5^ and odds ratio: 8.71 × 10^−2^) (Figure 1D). *PIK3CA* mutations are most frequently found in ER-positive tumours and has a strong correlation with estrogen receptor signalling. Further, a computational analysis based on gene signatures to score the individual patient samples to characterize their luminal and basal program further supported the hypothesis of the enrichment of luminal–basal hybrid cells in miR-18a/low tumours. Low levels of miR-18a leads to a less basal and more luminal phenotype in ER-negative tumours in both TCGA and METABRIC tumours (*p* < 0.0001) (Figure 1E). ER-negative tumours of our cohort were also stratified based on miR-18a expression into high (n = 24) and low (n = 30) groups based on the upper and lower quartiles of miR-18a expression. There was a significant negative correlation between miR-18a and *PGR* transcript, an estrogen-regulated gene (Pearson’s correlation co-efficient: −0.31, *p* = 0.02) (Figure 1F) in these tumours.

Cells with hybrid luminal/basal characteristics tend to be enriched for hybrid epithelial/mesenchymal and stemness traits. Hence, we examined the expression of stemness-associated protein integrin alpha 6/CD49f in MDA-MB-231/miR-18a/inh cells and the expression doubled on miR-18a inhibition (*p* = 0.0006) (Figure 1B). The cells were also examined for CD44 and CD24 expression as double positivity for CD44 and CD24 is a trait of hybrid E/M cells. The percentage of CD44^+^ CD24^+^ cells significantly increased on miR-18a inhibition by 9% (*p* = 0.03) (Figure 1G,H) in MDA-MB-468 and by 1% in MDA-MB-453 (*p* = 0.03) (Supplementary Figure S2). Since another characteristic trait of hybrid epithelial/mesenchymal cells is the dual positivity for Vimentin and E-cadherin, we evaluated the change in expression of these markers in MDA-MB-468/miR-18a/inh cells. There was a significant loss in the expression of E-cadherin (*p* = 0.05); however, the percentage of the dual positive Vimentin^+^ E-cadherin^+^ cells significantly increased after miR-18a inhibition (*p* = 0.03) (Figure 1I,J). This observation was further supported by the computational analysis based on gene signatures on the TCGA and the METABRIC series of tumours. Low miR-18a levels were associated with an increase in both epithelial (*p* < 0.005) and mesenchymal gene signatures (*p* = 0.0005) (Figure 1E).

### 3.2. ER-Negative Breast Cancer with Low miR-18a Is Associated with Low Proliferation and Enhanced EMT Traits

**Figure 2:**
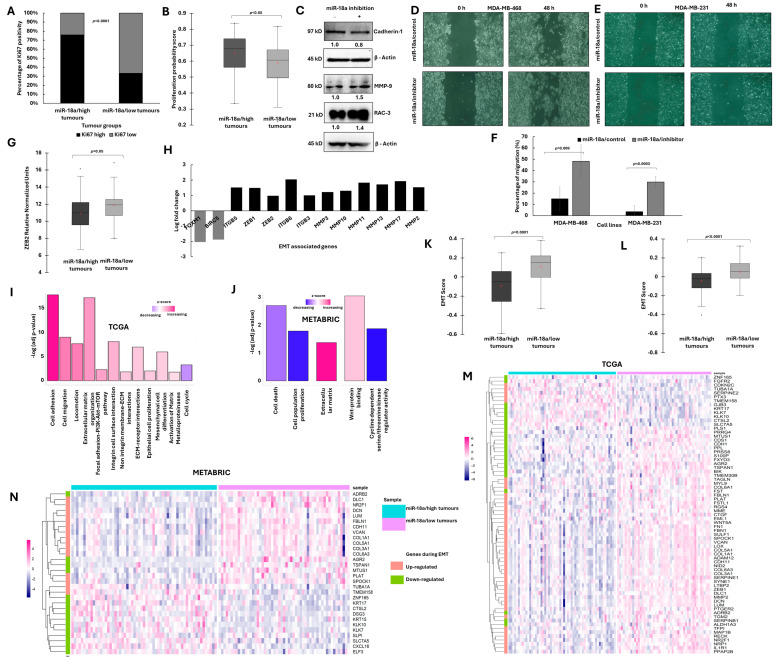
Association of reduced miR-18a levels with proliferation and EMT characteristics in ER-negative breast cancer. (**A**) Association of Ki67 index and miR-18a levels in miR-18a/high and miR-18a/low tumours and (**B**) association of proliferation probability score and miR-18a in miR-18a/high and miR-18a/low tumours of our cohort. (**C**) Change in protein expression levels of EMT-associated proteins in MDA-MB-231/miR-18a/cont and MDA-MB-231/miR-18a/inh cells. (**D**,**E**) Migratory ability as assessed by wound healing assay in MDA-MB-468/miR-18a/cont vs. MDA-MB-468/miR-18a/inh and MDA-MB-231/miR-18a/cont vs. MDA-MB-231/miR-18a/inh, respectively. (**F**) Percentage of migration from three independent trials in MDA-MB-468/miR-18a/cont vs. MDA-MB-468/miR-18a/inh and MDA-MB-231/miR-18a/cont vs. MB-231/miR-18a/inh. (**G**) Association of ZEB2 transcript levels and miR-18a in miR-18a/high and miR-18a/low tumours as assessed by q-PCR. (**H**) DEGs associated with EMT and cell proliferation in miR-18a/low, ER-negative tumours of TCGA. (**I**,**J**) Functional enrichment of DEGs depicting up-regulated and down-regulated pathways in miR-18a/low, ER-negative tumours of TCGA and METABRIC cohorts, respectively. (**K**,**L**) Evaluation of the levels of the EMT score derived from a pan-cancer 77 EMT gene signature in miR-18a/high and miR-18a/low, ER-negative tumours of TCGA and METABRIC cohorts, respectively. (**M**,**N**) Heat map depicting expression of genes up-regulated and down-regulated during the process of EMT (derived from EMT core list of 130 genes) in miR-18a/low and miR-18a/high groups of ER-negative tumours respectively of TCGA and METABRIC cohorts.

The ER-negative tumour samples of our breast cancer cohort were examined for the association of miR-18a with the Ki67 proliferation index (the Ki67 proliferation index was determined by immunohistochemistry as elaborated in the manuscript published previously) [24]. The tumours were stratified based on miR-18a levels; the tumours with less than the lower quartile expression of miR-18a (miR-18a/low) (n = 24) was compared with all the other tumours (miR-18a/high) (n = 81). Tumours with a Ki-67 index of 14 or more were considered as highly proliferative and the tumours with less than 14 were grouped as less proliferative. In total, 67% of the miR-18a/low tumours had a lower Ki67 expression when compared to 24% of the miR-18a/high tumours (*p* < 0.0001) (Figure 2A). The miR-18a levels were further used to correlate with a probability distribution of the tumour aggression score published previously [24] which was derived by fitting a binomial logistic regression model using two genes, *ANLN* and *BCL2*, as predictors and tumour grade 3 as the determinant. This score is referred to as the tumour aggression score. The miR-18a/low tumours were associated with a lower tumour aggression score (*p* < 0.05) (Figure 2B) when compared to the miR-18a/high tumours. This further suggests that miR-18a/low tumours enriched for hybrid E/M cells may be slow-proliferative and low-cycling than miR-18a/high tumours.

To further probe the role of miR-18a in the process of EMT, the levels of E-cadherin, MMP-9 and RAC3 were probed for in MDA-MB-231/miR-18a/inh cells. There was a significant loss of E-cadherin protein (up to 15%, *p* = 0.03) which is typically lost in epithelial cells to mark the beginning of contact inhibition and increased migration. Rac3 is another critical protein required to regulate adhesiveness and motility in breast cancer. The levels of Rac3 increased by 40% (*p* = 0.0008) and MMP9 levels increased by 55% (*p* = 0.002) (Figure 2C). We also observed an increase in the MMP9 levels in MDA-MB-453/miR-18a/inh cells by 48% (*p* = 0.05) (Supplementary Figure S3). Further, to confirm the increase in migratory ability, a wound healing assay was performed in both MDA-MB-231 and MDA-MB-468 cell lines. After miR-18a inhibition, migratory ability increased by 33% in MDA-MB-468 (*p* = 0.009) and by 26% in MDA-MB-231 (*p* = 0.0003) (Figure 2D–F). The observations were further confirmed using the ER-negative breast tumour specimens, (n = 105) of our cohort where the association of miR-18a with ZEB2, a master regulator of the EMT process, was evaluated. The miR-18a/low tumours segregated based on the lower quartiles of miR-18a expression were found to express high levels of *ZEB2* (*p* = 0.05) (Figure 2G) as assessed by q-PCR.

To evaluate these findings in a larger cohort of tumours, the ER-negative tumours of the TCGA and the METABRIC cohort were stratified as miR-18a/low and miR-18a/high as described in the methods. Analysis of the DEGs in TCGA tumours revealed that the miR-18a/low tumours expressed high levels of EMT master regulators-*ZEB1* and *ZEB2* and Matrix metalloproteinases, *MMP2*, *MMP3*, *MMP10*, *MMP11*, *MMP13* and *MMP17* (*p* < 0.05). The level of *ITGB5*, a glycoprotein involved in facilitating cell migration and angiogenesis, was also highly expressed in these tumours (*p* < 0.05) (Figure 2H). METABRIC miR-18a/low tumours displayed elevated levels of *MMP2* (*p* < 0.05). *TWIST1* was also highly expressed by miR-18a/low tumours of both TCGA and METABRIC cohort (Supplementary Figure S4). The level of cell proliferation-associated genes, *FOXM1* and *BIRC5* genes, was expressed less in the miR-18a/low tumours (*p* < 0.05) (Figure 2H). Both FOXM1 and BIRC5 are implicated in driving tumour progression by increasing the cell proliferation rates [39,40]. This result is indeed a reflection of the earlier results observed in our series of tumours where miR-18a/low tumours correlated with a lower Ki67 index.

Functional enrichment of differentially expressed genes (DEGs) in miR-18a/low tumours demonstrated up-regulation of the pathways related to cell motility and migration, ECM activation, pathways related to activation of matrix metalloproteases, Wnt signalling and focal adhesion-PI3K-Akt signalling in TCGA (Figure 2I) and METABRIC series (Figure 2J) (*p* < 0.05). Moreover, pathways related to cell proliferation and cell cycle were down-regulated in these tumours (*p* < 0.05) (Figure 2I,J). Analysis of the tumours using a pan-cancer 77 EMT gene signature derived from 11 cancer subtypes showed that miR-18a/low tumours of both TCGA and METABRIC series were associated with a higher EMT score (*p* < 0.0001) (Figure 2K,L). Association of miR-18a levels was also examined with a core gene list of 130 EMT-related genes derived from a meta-analysis as described in the methods. In the majority of miR-18a/low tumours, there was a higher expression of the EMT-related genes that are up-regulated as part of the 130 EMT core gene list and lower expression of genes that are down-regulated in the EMT core gene list. (Figure 2M,N).

### 3.3. Low Levels of miR-18a Are Associated with Increased Chemoresistance and Cancer Stemness in the ER-Negative Subtype

**Figure 3:**
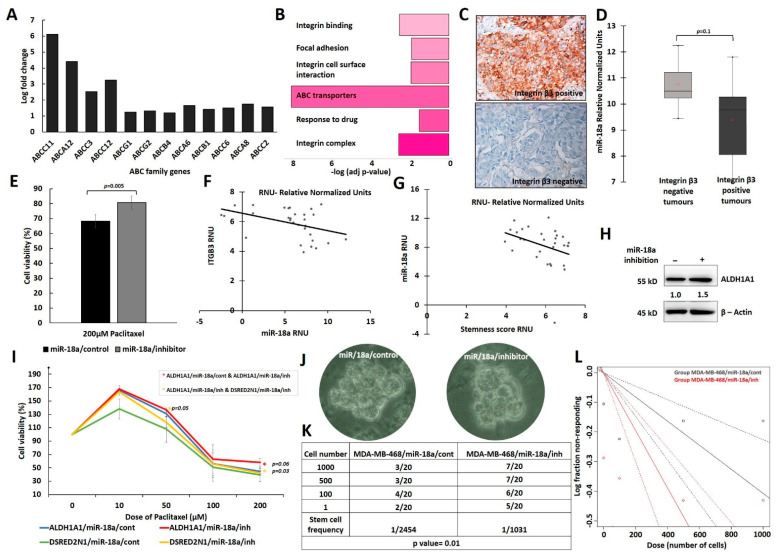
Association of low miR-18a levels with drug response and stemness acquisition in ER-negative breast cancer. (**A**) Fold change of DEGs associated with drug transporter gene family (ATP-binding cassette transporter) in miR-18a/low, ER-negative tumours of TCGA cohort. (**B**) Functional enrichment of DEGs depicting up-regulated pathways in miR-18a/low, ER-negative tumours of TCGA. (**C**) Representative IHC images of Integrin β3-stained sections of residual tumours post-NACT. (**D**) Association of Integrin β3 protein and miR-18a in ER-negative residual tumours post-NACT. (**E**) Cell viability of MDA-MB-468/miR-18a/cont and MDA-MB-468/miR-18a/inh after 200 µM paclitaxel treatment. (**F**) Association of *ITGB3* and miR-18a transcript levels and (**G**) association of stemness score and miR-18a transcript levels in miR-18a/low treatment naïve primary ER-negative tumours as assessed by q-PCR. (**H**) Change in expression levels of ALDH1A1 between MDA-MB-468/miR-18a/cont and MDA-MB-468/miR-18a/inh. (**I**) Percentage of cell viability of ALDH1A1/miR-18a/cont, ALDH1A1/miR-18a/inh, DsRed2N1/miR-18a/cont and DsRed2N1/miR-18a/inh groups in MDA-MB-231 after paclitaxel treatment across doses ranging from 10–200 µM. (**J**) Representative images of spheres obtained from MDA-MB-468/miR-18a/cont and MDA-MB-468/miR-18a/inh cells. (**K**) Table depicting clonogenicity (stem cell frequency) calculated from extreme limiting dilution assay. (**L**) Graph depicting clonogenicity from extreme limiting dilution assay performed on MDA-MB-468/miR-18a/cont and MDA-MB-468/miR-18a/inh groups across the same initial seeding dose range of 1–1000 cells.

ABC (ATP-binding cassette) proteins are known to contribute to cancer progression through detoxification of drugs and xenobiotics. In addition to this, aberrant expression of these ABC proteins is known to stimulate the hallmarks of cancer and drive the pathways necessary for tumour progression. There are several human ABC proteins, which are classified into seven families from A to G based on sequence homology [41,42]. We checked the gene expression levels of 12 of these proteins and checked their association with miR-18a levels. Analysis of the genes that are differentially expressed in miR-18a/low tumours of the TCGA dataset revealed an increased expression of the genes, namely *ABCC11*, *ABCA12*, *ABCC3*, *ABCC12*, *ABCG1*, *ABCG2* etc. (*p* < 0.05) (Figure 3A). *ABCC11* and ABCC12 were highly expressed in miR-18a/low tumours of the METABRIC dataset (Supplementary Figure S4). Functional enrichment of these DEGs demonstrated up-regulation of the pathways related to drug response, drug transporter genes, integrin cell surface interactions, integrin binding and focal–adhesion complex (*p* < 0.05) (Figure 3B). We have shown previously that high integrin β3 levels in triple negative breast cancer contributed to chemoresistance by leading to the repression of BAD [20]. As integrins are known to trigger downstream pro-survival signalling cascades, we looked at the protein expression of integrin β3 by immunohistochemistry in the residual tumours post-neoadjuvant chemotherapy from partial and non-responders. Integrin β3 protein was expressed by the endothelial cells, stromal immune cells, smooth muscle cells, tumour and peritumoural cells in the residual tumour sections. Figure 3C shows representative IHC images for the expression of integrin β3. The association of miR-18a in integrin β3 negative and positive tumours were probed for in ER-negative residual tumours (n = 13). miR-18a/low tumours expressed higher levels of integrin β3 (*p* = 0.1) when compared to miR-18a/high tumours (Figure 3D).

Drug resistance was then measured after inhibition of miR-18a in MDA-MB-468, with paclitaxel at varying doses from 10 µM to 200 µM. The drug sensitivity of MDA-MB-468/miR-18a/inh was not different from MDA-MB-468/miR-18a/cont at various doses from 10 µM to 100 µM (*p* > 0.05). With 200 µM paclitaxel treatment, there was a 12% increase in the cell viability of MDA-MB-468/miR-18a/inh when compared to MDA-MB-468/miR-18a/cont (*p* = 0.005) (Figure 3E). The Selectivity Index calculated was 58% for MDA-MB-468/miR-18a/inh and 72% for MDA-MB-468/miR-18a/cont (*p* = 0.2) (Supplementary Figure S5a).

Cells with hybrid luminal/basal–epithelial/mesenchymal features tend to display enhanced cancer stem cell (CSC) properties [43]. Integrin β3/CD61 is also identified as a mammary progenitor marker that identifies the cancer stem cell population enriched for tumorigenic potential [44]. The association of miR-18a/low tumours with integrin β3 in post-neoadjuvant residual tumours intrigued us to probe for the same association in primary treatment naïve tumours of our cohort. ER-negative tumours of our cohort were also stratified based on miR-18a lower quartile expression into miR-18a/low (n = 30) tumours. Within miR-18a/low tumours, there was a significantly negative correlation between *ITGB3* and miR-18a (Pearson’s correlation co-efficient: −0.42, *p* = 0.02) (Figure 3F). To examine other features of cancer stemness displayed by miR-18a/low tumours, q-PCR assay for cancer stemness associated genes, *SALL4*, *LGR5*, *BMPR1B*, was performed in these tumours. A stemness score was arrived at by calculating the mean gene score of *SALL4, LGR5*, *BMPR1B* and *ITGB3*. Within miR-18a/low tumours, there was a negative association between miR-18a and the stemness score (Pearson’s correlation co-efficient: −0.33, *p* = 0.06) (Figure 3G), implying that as miR-18a levels reduced, the stemness score was higher in these ER-negative tumours. The levels of *ALDH1A1* and *BMP4* were also high in the METABRIC miR-18a/low tumours on analysis of the DEGs (*p* < 0.05) (Supplementary Figure S4). BMP4 is implicated in promoting metastasis in breast cancer by enhancing cancer stemness. On miR-18a inhibition in MDA-MB-468, we also observed an increase in the levels of ALDH1A1 by 50% (*p* = 0.04) (Figure 3H).

To further examine the effects of miR-18a inhibition on cells enriched for cancer stemness, MDA-MB-231 cells transfected with ALDH1A1-DsRed2N1 plasmid was used. miR-18a was inhibited in MDA-MB-231 cells with ALDH1A1-DsRed2N1 reporter and the control (DsRed2N1) cells using the microOFF™ inhibitor and inhibitor negative control as described in the methods. To confirm the stemness of ALDH1A1-DsRed2N1 cells, the level of stemness-associated genes *LGR-5* and *SOX2* were assessed by q-PCR and were found to be higher than control (DsRed2N1) cells (Supplementary Figure S5b). Post-transfection, the cells were subjected to drug sensitivity assays with paclitaxel with varying doses from 10 µM to 200 µM. ALDH1A1/miR-18a/inh cells were more resistant (by 14%) than ALDH1A1/miR-18a/cont only at a 200 µM dose of paclitaxel (*p* = 0.06). However, ALDH1A1/miR-18a/inh cells were significantly more chemo-resistant than DSRED2N1/miR-18a/inh at both 50 µM (by 18%; *p* = 0.05) and 200 µM (by 16%; *p* = 0.03) doses of paclitaxel (Figure 3I). Although we did not observe an overwhelming effect on chemoresistance after miR-18a inhibition in the cell lines, inhibition of miR-18a in stem-like cells over-expressing ALDHA1 led to an increase in chemoresistance, an indication that low miR-18a levels in cells with stem-like attributes may convert them into a more chemo-resistant phenotype.

To further confirm the role of low miR-18a in rendering stemness attribute to breast cancer cells, mammosphere-forming ability was evaluated in MDA-MB-468 and MDA-MB-453. MDA-MB-468/miR-18a/cont and MDA-MB-453/miR-18a/cont formed a lesser number of distinct spheres than MDA-MB-468/miR-18a/inh and MDA-MB-453/miR-18a/inh (Figure 3J). The spheres were serially propagated, and extreme limiting dilution assay was performed to assess the clonogenicity of the spheres formed. The clonogenicity (1/stem cell frequency) was 1/1031 in MDA-MB-468/miR-18a/inh cells and almost two times lower in MDA-MB-468/miR-18a/cont (1/2454) cells (*p* = 0.01) (Figure 3K,L). The clonogenicity (1/stem cell frequency) was 1/233 in MDA-MB-453/miR-18a/inh cells and approximately four times lower in MDA-MB-453/miR-18a/cont (1/929) cells (*p* < 0.001) (Supplementary Figure S6).

### 3.4. Lower Levels of miR-18a in ER-Negative Breast Cancer Correlates with Increased Stromal-Immune Infiltration and Immunosuppression

**Figure 4:**
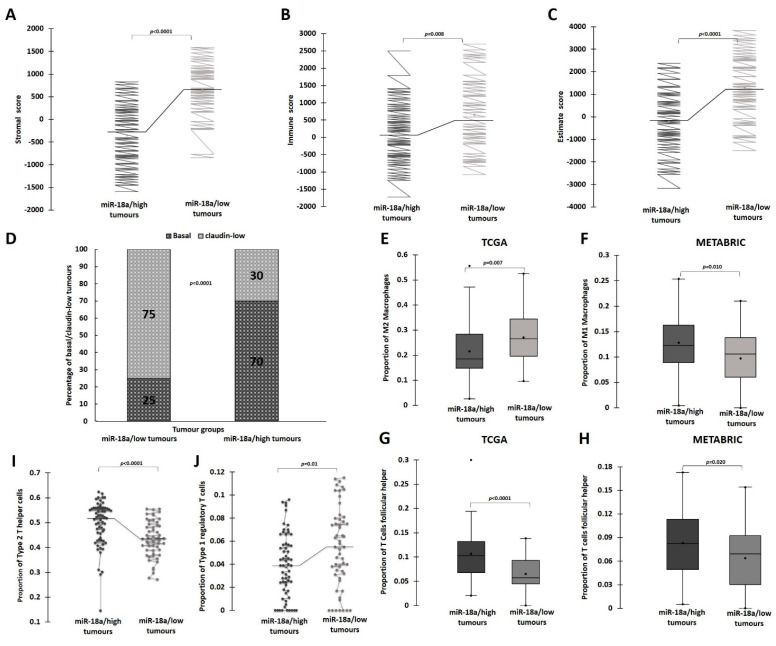
Correlation of low miR-18a levels with immune suppression in ER-negative breast cancer. (**A**–**C**) ESTIMATE-generated Stromal score, Immune score and Estimate score for miR-18a/high and miR-18a/low tumours of TCGA cohort. (**D**) Graph representing proportion of claudin-low and basal tumour subtypes in miR-18a/low and miR-18a/high, ER-negative tumours of METABRIC series. (**E**,**F**) CIBERSORT analysis depicting the proportions of M2 and M1 macrophages in miR-18a/high and miR-18a/low tumours. (**G**,**H**) CIBERSORT analysis depicting the proportions of T follicular helper cells in miR-18a/high and miR-18a/low, ER-negative tumours of TCGA and METABRIC cohorts, respectively. (**I**,**J**) ImmuneCellAI analysis depicting the proportions of Th2 (Type 2 helper cells) and Tr1 (Type 1 regulatory T cells) in miR-18a/high and miR-18a/low, ER-negative tumours of TCGA series respectively.

A bioinformatic approach was followed to estimate the proportion of immune infiltrate in miR-18a/low tumours of the TCGA and the METABRIC cohort as described in the methods. The ESTIMATE (Estimation of Stromal and Immune cells in Malignant Tumour tissues using Expression data) is a tool that uses gene expression data for predicting tumour purity, and the presence of infiltrating stromal/immune cells in tumour tissues. The ESTIMATE score generates three different scores. The stromal score depicts the stromal presence in these tumours and the immune score captures the immune infiltration in the tumours. These two scores form the basis of the ESTIMATE score that is an inference of the tumour purity. The miR-18a/low tumours were associated with a higher stromal (*p* < 0.0001) (Figure 4A) and immune score (*p* = 0.008) (Figure 4B). These tumours also had a higher ESTIMATE score when compared to miR-18a/high tumours (*p* < 0.0001) (Figure 4C).

Most of the traits we have identified as associated with miR-18a/low tumours overlap with that of the claudin-low tumours. The claudin-low subtype represents ER-negative tumours that express EMT- and stemness-associated genes. They are also known to have marked stromal and immune infiltration. They are associated with a lower proliferation rate and a lower Ki-67 index when compared to non-claudin-low tumours [45]. Tumours from the METABRIC series were used to examine the proportion of claudin-low and basal tumours in the miR-18a/high and low tumours. When only basal and claudin-low tumours are grouped together, 21/28 of miR-18a/low tumours represent the claudin-low subtype when compared to only 18/60 of miR-18a/high tumours (*p* < 0.0001) (Figure 4D).

Further, to examine if the immune–stromal infiltration in miR-18a/low tumours was suggestive of an immunosuppressive microenvironment, immune cell identification was performed using CIBERSORT analysis, a method that describes the cell composition of complex tissue from their gene expression profiles in tumours. The analysis in both the cohorts revealed that ER-negative tumours with low miR-18a correlated with increased proportions of M2 macrophages (TCGA—*p* = 0.007) and decreased proportion of M1 macrophages (METABRIC—*p* = 0.01) (Figure 4E,F). miR-18a/low tumours also had a significantly lower presence of T-follicular helper cells (TCGA—*p* < 0.0001, METABRIC—*p* = 0.02), which are specialised T cells that play a crucial role in protective immunity by helping B cells [46] (Figure 4G,H). Further evaluation of the immune composition was performed using ImmuCellAI, a gene expression-based method for estimating the abundance of multiple types of T-cell subsets, in the TCGA cohort. miR-18a/low tumours had a lower proportion of Th2 (Type 2 helper cells) (*p* < 0.0001) and a higher proportion of Tr1 (Type 1 regulatory T cells) (*p* = 0.01) (Figure 4I,J). Th2 cells participate in building anti-tumour immunity and aid in tumour clearance and Tr1 mediate immune suppression and establish peripheral tolerance. The differential expression of M1 macrophages in TCGA and differential expression of M2 macrophages, Type 2 helper and Type-1 regulatory cells in METABRIC datasets did not emerge as statistically significant (Supplementary Figure S7a).

### 3.5. HIF-1α Inhibition Leads to a Reversal of Hybrid E/M Phenotype in miR-18a Inhibited Cells

**Figure 5:**
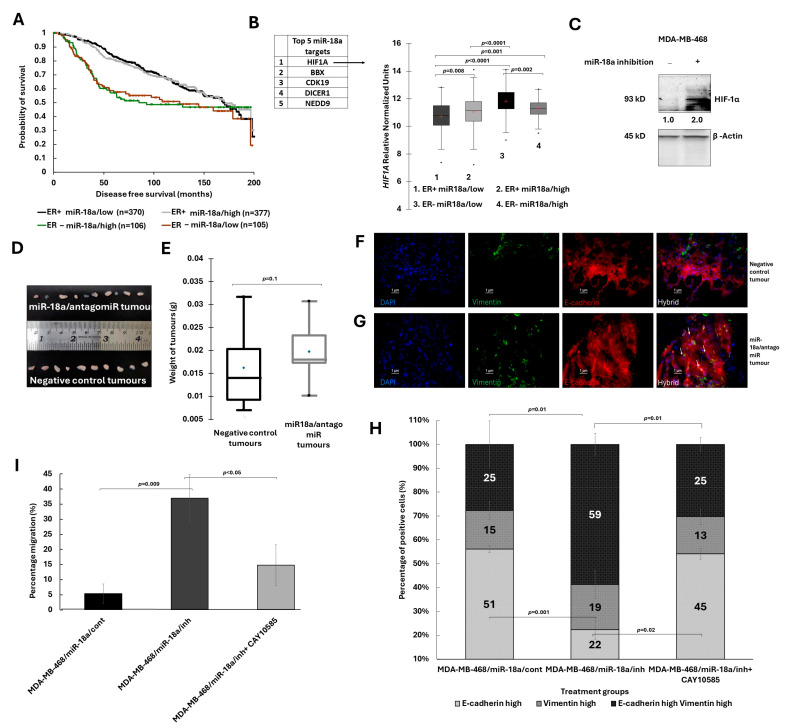
Effect of miR-18a inhibition on mice-induced tumours in vivo and association with *HIF1A* expression. (**A**) Kaplan–Meier analysis depicting disease-free survival in ER-positive/miR-18a/low, ER-positive/miR-18a/high, ER-negative/miR-18a/low and ER-negative/miR-18a/high tumours of the METABRIC cohort. (**B**) Expression of *HIF1A* in ER-positive/miR-18a/low, ER-positive/miR-18a/high, ER-negative/miR-18a/low and ER-negative/miR-18a/high tumours of the TCGA cohort. (**C**) Increased expression levels of HIF-1α in MDA-MB-468/miR-18a/inh in comparison to MDA-MB-468/miR-18a/cont. (**D**) Images of tumours harvested from mice injected with miR-18a/antagomiR cells and antagomiR negative control cells post-21-days of injection. (**E**) Box plot depicting the average weight of the tumours harvested from miR-18a/antagomiR and the antagomiR negative control mice. (**F**,**G**) Immunofluorescence to demonstrate the dual positivity of E-cadherin and Vimentin in tumour sections from miR-18a/antagomiR tumour and absence of dual positivity of E-cadherin and Vimentin in antagomiR negative control tumour. (**H**) Quantitative analysis of immunofluorescence for expression of Vimentin and E-cadherin in MDA-MB-468/miR-18a/cont, MDA-MB-468/miR-18a/inh and MDA-MB-468/miR-18a/inh + CAY10585. (**I**) Percentage of migration as measured by wound healing assay in MDA-MB-468/miR-18a/cont, MDA-MB-468/miR-18a/inh and MDA-MB-468/miR-18a/inh + CAY10585.

To further decipher the clinical relevance and the prognostic implication of low miR-18a levels in ER-negative breast cancer, a Kaplan–Meier survival analysis was performed on the METABRIC series of samples. Tumours were stratified based on the median expression levels of miR-18a in both ER-negative (median—8.2) and ER-positive tumour samples (median—7.3). The analysis was then performed between ER-positive/miR-18a/low (n = 370), ER-positive/miR-18a/high (n = 377), ER-negative/miR-18a/low (n = 105) and ER-negative/miR-18a/high (n = 106) tumour samples (Figure 5A). There was no significant difference in disease-free survival between the ER-negative/miR-18a/low tumours and the ER-negative/miR-18a/high tumours (*p* = 0.9). However, the disease-free survival was significantly different between the ER-positive/miR-18a/low tumours vs. ER-negative/miR-18a/low tumours (*p* = 0.001) and the ER-positive/miR-18a/high tumours vs. ER-negative/miR-18a/low tumours (*p* = 0.001). The mean survival time for ER-negative/miR-18a/low tumours was 114 months when compared to 141 months in ER-positive/miR-18a/low tumours.

A target scan analysis was then performed to decipher the gene targets of miR-18a that may be differentially expressed and driving the effects brought about by the low miR-18a levels in the miR-18a/low tumours of the ER-negative breast cancer subtype. Fifteen targets identified by at least three different target mining software were shortlisted (Table 1). The expression levels of these targets were analysed in the tumours of the TCGA by stratifying both ER-positive and ER-negative tumours into two groups each based on upper and lower quartiles of miR-18a expression. Of all the targets, *PDE4D* and more significantly, *HIF1A* showed high expression in ER-negative miR-18a/low tumours (Figure 5B). *HIF1A* emerged as the target predicted by all target prediction tools and miR-18a dependent HIF-1α and hypoxic regulation has already been reported in basal breast cancer previously [18]. Hence, the levels of HIF-1α protein were probed in MDA-MB-468/miR-18a/inh and MDA-MB-231/miR-18a/inh cells. There was only a marginal increase in the HIF-1α levels in MDA-MB-231/miR-18a/inh cells (Supplementary Figure S7b); however, the expression doubled in MDA-MB-468 post-miR-18a inhibition (*p* = 0.05) (Figure 5C). This increase in the HIF-1α levels prompted us to examine the presence of an activated hypoxic gene expression, if any. Genes involved in hypoxia were retrieved using literature mining [47,48,49] and were mapped to TCGA dataset. The genes which were showing a significant difference (*p* < 0.05) between miR-18a/high and low tumours were filtered. The heatmap representing the pattern of expression of filtered hypoxia genes shows that the hypoxia-related genes were up-regulated in the miR-18a/low tumours (Supplementary Figure S7c).

To further evaluate the tumourigenic potential of miR-18a-inhibited cells, in vivo xenograft experiments were performed by inducing orthotopic tumours in NSG/NOD-SCID mice. The tumours formed with miR-18a/antagomiR cells were larger in volume by 21% when compared to tumours formed from antagomiR negative control cells (*p* = 0.1) (Figure 5D,E). miR-18a/antagomiR cells formed a distinct mass of tumour. However, the negative control cells formed only small bud of tumours in the mouse mammary glands as evident from the H&E analysis of the tumour sections (Supplementary Figure S7d). Immunofluorescence staining was performed on the tumour sections from the miR-18a/antagomiR tumours and the antagomiR negative control tumours for the markers Vimentin and E-cadherin. miR-18a/antagomiR tumours showed a higher proportion of cells co-expressing Vimentin and E-cadherin (Figure 5F,G and Supplementary Figure S7e) when compared to negative control tumours. The results further support the hypothesis that low levels of miR-18a drive tumour progression by enriching for hybrid E/M cells in ER-negative breast cancer.

Further to confirm the role of HIF-1α in miR-18a-inhibited cells, the HIF-1α pathway was blocked using CAY10585, a HIF-1α inhibitor that suppresses transcription of HIF-1α target genes. We observed that the percentage of hybrid E/M cells (Vimentin^+^ E-cadherin^+^) reduced by 30% (*p* = 0.01) accompanied by an increase in the proportion of cells that were Vimentin–E-cadherin^+^ (*p* = 0.02) (Figure 5H). Moreover, the migratory ability of the MDA-MB-468/miR-18a/inh cells was found to decrease by 22% with HIF-1α pathway inhibition (*p* < 0.05) (Figure 5I). The results show the existence of a possible HIF-1α-dependent pathway regulated by low levels of miR-18a in the ER-negative subtype driving the hybrid E/M phenotype.

## 4. Discussion

The complexity of the ER-negative subtype of breast cancer arises due to the heterogeneous nature of the disease and this poses a challenge to effective treatment and eventually the prognosis of the patients [5,6]. The ER-negative subtype is usually more aggressive and has a worse prognosis than the ER-positive subtype. A better understanding of the disease exists with the advancement in genomics; however, the ER-negative subtype has very few targeted and tailored therapy options [50]. The molecular subtyping studies, especially the PAM50 classification, have unravelled the existence of basal-like and Her2-enriched subclasses within the ER-negative subtype [51].

Genome sequencing and mutational profiling conducted by the TCGA network and multiple other groups have led to the identification of mutational signatures that not only affect genomic signatures but also bring about epigenetic changes [5,7]. This may be causal for tumour heterogeneity leading to differential activation of signalling pathways that eventually lead to divergent molecular signatures. More reports emerge with evidence of a single miRNA playing tumour-promoting roles and tumour-suppressive roles among cancer subtypes [15,52]. We have previously reported the tumour-promoting role of high levels of miR-18a that leads to Wnt pathway activation, thus promoting metastasis and poor prognosis in ER-positive breast cancer. We have also identified that miR-18a-driven Wnt pathway activation may be the basis for the ‘immune cold’ phenotype that is displayed by ER-positive tumours [16,17]. In this manuscript, we present evidence for atypical biology that may be driven by low levels of miR-18a in the absence of expression of the hormone receptors ER and PR.

The ER-negative tumours of our cohort expressed higher levels of miR-18a when compared to ER-positive tumours. The high levels of miR-18a expression and its effect on prognosis in triple-negative breast cancers have been reported previously [18,19]. Nevertheless, the tumours that expressed lower levels of this miRNA within the ER-negative subtype were found to retain a different biology. These tumours were found to have both epithelial and mesenchymal traits thus exhibiting the traits of hybrid E/M tumours. In addition, they were found to have increased luminal traits also making them luminal/basal hybrid tumours. In vitro and computational analysis further confirmed these findings. The miR-18a/low tumours were associated with a lower Ki-67 index, lower proliferation rates and features of increased migration and EMT. ALDH1A1 levels increased post-inhibition of miR-18a and this led to an increase in drug resistance. Inhibition of miR-18a also increased clonogenicity and mammosphere-forming ability. In silico analysis also showed a correlation of low miR-18a levels to immunosuppression in ER-negative tumours.

It was also intriguing that a large proportion of miR-18a/low tumours overlapped with the claudin-low tumours. These findings are clinically relevant as the claudin-low is a much-uncharacterised subtype of breast cancer. We did not observe any difference between the prognosis of miR-18a/low and miR-18a/high tumours of the ER-negative subtype. This may be attributed to the heterogeneity in gene expression patterns and mutational profiles generally noted in ER-negative tumours. Biological pathways altered in ER-negative tumours are complex and varied due to the heterogeneity of these tumours being an admixture of triple negative, HER2-amplified and claudin-low-like tumours. ER is central to the biology of breast cancer and our findings confer roles played by miR-18a in ER-negative tumours through ER regulation. Interestingly, in in vivo models, miR-18a-inhibited cells formed larger tumours and they expressed more hybrid E/M cells.

The ER-negative tumours considered for analysis comprised of tumours featured by the absence of ER expression and presence/absence of the HER2 growth factor. On performing the various in silico analysis after stratification of the miR-18a/low tumours based on HER2 status, the changes in phenotype observed in miR-18a/low tumours were similar in both the groups. This is an indication that miR-18a plays a similar role in both TNBC and ER-HER2+ tumours (Supplementary Figures S8–S10).

Regulation of a miR-18a-mediated hypoxic gene signature by activation of HIF-1α in basal breast cancer has been reported previously [18]. We also observed that the miR-18a/low tumours expressed high levels of *HIF1A*. HIF-1α inhibition using a small molecule in miR-18a-inhibited cells brought down the proportion of hybrid E/M cells and the migratory ability. The results mirror the observations reported previously where it was noted that the maintenance of a highly tumourigenic E/M hybrid state was brought about by activation of EMT-inducing transcription factors and canonical Wnt signalling in basal breast cancer cells. The role of hypoxia-driven signalling through the activation of P4HA2 in maintaining the partial or hybrid E/M phenotype in breast cancer was also recently examined [53,54].

miR-18a regulates ER signalling by binding to the 3′UTR and regulating its expression and this may be one of the ways by which epigenetic silencing of ER is mediated during the evolution of ER-negative tumours [55]. Higher levels of miR-18a in ER-negative tumours may be an implication of such a regulation. However, lower levels of miR-18a in these tumours may be activating pathways required for the enrichment of hybrid E/M cells that lead to a multitude of phenotype changes. Recent studies render functionalities of gene expression tuning and expression buffering to miRNAs. Expression buffering is a process by which weakening in the variance of the expression level of the target genes is mediated by miRNAs [52]. The biology seen in miR-18a/low tumours may be attributed to such a buffering function where low levels fail to buffer the mean levels of miR-18a target genes such as *HIF1A* and thus lead to alternate phenotypic changes. The blocking of HIF-1α and reversal of hybrid E/M phenotype is a confirmation of such altered buffering. The novelty of the study includes the unravelling of the novel association of low miR-18a levels and the enrichment of hybrid E/M cells that leads to phenotypic changes including that of increased migration and stemness in a subgroup of ER-negative tumours that may be HIF-1α driven.

Limitations of this study include a smaller sample size of miR-18a/low ER-negative tumours used for the analysis. However, the fact that we were able to verify the observations in two larger datasets increases the credibility of the observations made in clinical specimens. In vitro results further validate and strengthen our results and observations. The immune suppression phenotype observed was identified using only an in silico approach. Further, in vitro validations need to be performed to confirm the immunosuppressive effects of miR-18a in ER-negative tumours. Since the discovery of miRNAs in the field of cancer, there also has been an increase in the focus on the therapeutic implications of these small molecules. miRNA-based anti-cancer therapeutics are currently being developed with the goal of improving disease-free survival in cancer [56]. The main benefit of employing anti-miRNA-based strategies is based on the concept that multiple effectors of various signalling pathways can be targeted. Strategies that include the use of antisense oligonucleotides and targeted nanoparticle therapy have emerged as very promising for personalised treatment in cancer [57,58]. Multiple studies have recently emerged that have used nanocarriers for the successful targeted delivery of microRNAs [59,60,61]. The pleiotropic and multifaceted nature of these small regulatory molecules makes them attractive drug targets and amenable to tweaking, especially for the ER-negative subtype that is vastly heterogeneous. Stratifying these tumours based on epigenetic phenotypic alterations can become a promising strategy for personalised medicine.

## Figures and Tables

**Table 1:** List of miR-18a targets: Target scan analysis was performed to decipher the gene targets of miR-18a. Fifteen targets identified by at least three different target mining softwares and their known function are enlisted below.

Sl No.	Gene	Function (Source—GeneCards, NCBI Gene)
1	*HIF1A*	Mediates hypoxia-induced expression of mRNA-encoding genes; regulates the expression of non-coding RNAs, which are critical regulators of migration, invasion and metastasis
2	*BBX*	Transcription factor necessary for cell cycle progression from G1 to S phase
3	*CDK19*	Mediator kinases, transcriptional co-regulators
4	*DICER1*	Responsible for cleaving double-stranded RNAs into small interfering RNAs and microRNAs
5	*NEDD9*	Positive regulator of epithelial–mesenchymal transition and promotes invasion
6	*EPB41L1*	Role in cell adhesion and migration, malignant progression
7	*ESR1*	Regulates the transcription of estrogen-inducible genes that play a role in growth, metabolism, sexual development, gestation
8	*GLRB*	Down-regulation of neuronal excitability, generation of inhibitory postsynaptic currents
9	*INADL*	Mediate protein–protein interactions, regulate the formation and stabilization of tight junctions
10	*MAP3K1*	Serine/threonine kinase in multiple cell signalling cascades
11	*PDE4D*	Major regulators of cAMP-hydrolyzing activity
12	*PHC3*	Transcriptional repression, chromatin remodelling and modification of histones
13	*RORA*	Interacts with NM23-2, a nucleoside diphosphate kinase involved in organogenesis and differentiation, as well as with NM23-1, the product of a tumour metastasis suppressor candidate gene
14	*SH3BP4*	Involved in cargo-specific control of clathrin-mediated endocytosis, specifically controlling the internalization of a specific protein receptor.
15	*ZNF367*	Transcriptionally activates KIF15 and regulates cell cycle

## Data Availability

The hyperlinks to publicly archived datasets analysed or generated during the study have been reported in the article where applicable.

## Supplementary Materials

The following supporting information can be downloaded at: https://www.mdpi.com/article/10.3390/cells13100821/s1, Figure S1: Expression level of *BIRC3*, *HIF1A*, *DICER* and *CDK19* after miR-18a inhibition as measured by q-PCR; Figure S2: Quantitative assessment of CD44 and CD24 expression in MDA-MB-453/miR-18a/cont and MDA-MB-453/miR-18a/inh as assessed by flow cytometry; Figure S3: Change in protein expression levels of MMP9 levels in MDA-MB-453/miR-18a/cont and MDA-MB-453/miR-18a/inh cells; Figure S4: DEGs associated with EMT and drug resistance in miR-18a/low, ER-negative tumours of METABRIC; Figure S5a: The SI index in percentage calculated for MDA-MB-468/miR-18a/inh when compared to MDA-MB-468/miR-18a/cont (calculated with reference to MCF10A); Figure S5b: Expression level of stemness genes in ALDH1A1-DsRed2N1 cells vs DsRed2N1 cells; Figure S6: Representative images of spheres obtained from MDA-MB-453/miR-18a/cont and MDA-MB-453/miR-18a/inh cells and table and graph depicting clonogenicity (stem cell frequency) calculated from extreme limiting dilution assay performed on MDA-MB-453/miR-18a/cont and MDA-MB-453/miR-18a/inh groups across the same initial seeding dose range of 1–1000 cells; Figure S7a: CIBERSORT analysis depicting the proportions of M1 and M2 macrophages in miR-18a/high and miR-18a/low tumours of TCGA and METABRIC cohorts respectively, immune cell AI analysis depicting proportion of Type 2 T helper cells and Type 1 regulatory T cells in the METABRIC cohort. Figure S7b: Change in protein expression levels of HIF-1α levels in MDA-MB-231/miR-18a/cont and MDA-MB-231/miR-18a/inh cells. Figure S7c: The heatmap representing the pattern of expression of filtered hypoxia genes in the miR-18a/low tumors of TCGA; Supplementary Figure S7d: Representative H&E stained image of tumour from mice injected with miR-18a/antagomiR and and antagomiR negative control cells; Figure S7e: Change in protein expression levels of E-cadherin and Vimentin levels in MDA-MB-231/miR-18a/cont and MDA-MB-231/miR-18a/inh cells; Figure S8: Mutational spectrum analysis depicting higher *PIK3CA* mutation load in both TNBC and ER-HER2+ tumors of the miR-18a/low ER-negative tumours of METABRIC cohort; Figure S9: Partial EMT score between the TNBC and ER-HER2+ tumors of the miR-18a/low ER-negative tumours of TCGA and METABRIC cohort.; Figure S10: 77 gene EMT Score between the TNBC and ER-HER2+ tumors of the miR-18a/low ER-negative tumours of the TCGA and METABRIC cohort; Table S1:List of antibodies and their dilutions; Table S2: Clinico-pathological characteristics of 211 ER-negative patients used for analysis from TCGA dataset; Table S3: Clinico-pathological characteristics of 265 ER-negative patients used for analysis from METABRIC dataset; Table S4: List of luminal and basal genes; Table S5: Clinico-pathological characteristics of 105 ER-negative patients used for analysis from our case series; Table S6: Clinical characteristics of post NACT residual tumors: Clinico-pathological characteristics of (n = 54) post NACT residual tumors and (n = 43) tumors with adequate tissue available for estimation of integrin β3 and miR-18a; Table S7: List of primer sequences used in q-RT-PCR analysis.

## Abbreviations

E/M	Epithelial/Mesenchymal
miRNAs	microRNAs
ELDA	Extreme limiting dilution assay
EMT	Epithelial to Mesenchymal Transition
E/M hybrid	Epithelial–Mesenchymal Hybrid
EMP	Epithelial–Mesenchymal Plasticity
NCCS	National Centre for Cell Science
ATCC	American Type Culture Collection
PFA	Paraformaldehyde
FFPE	Formalin-fixed paraffin-embedded
QC	Quality control
NACT	Neoadjuvant chemotherapy
IHC	Immunohistochemistry
DEGs	Differentially Expressed Genes
FGF	Fibroblast growth factor
EGF	Epidermal growth factor
CSC	Cancer stem cell
ESTIMATE	Estimation of Stromal and Immune cells in Malignant Tumour tissues using Expression data
