# Supplementary material for: Elucidating the Role of MicroRNA-18a in Propelling a Hybrid Epithelial–Mesenchymal Phenotype and Driving Malignant Progression in ER-Negative Breast Cancer
Source: Cells. 2024 May 10;13(10):821. doi: 10.3390/cells13100821 (PMC11119613; doi:10.3390/cells13100821)

Elucidating the role of microRNA-18a in propelling a hybrid epithelial-mesenchymal phenotype and driving malignant progression in ER-negative breast cancer

Madhumathy G Nair[1#], Apoorva D Mavatkar[1*], Chandrakala M Naidu [1*], Snijesh VP[1], Anupama CE[1], Savitha Rajarajan[1], Sarthak Sahoo[2], Gayathri Mohan[3], Vishnu Sunil Jaikumar [4], Rakesh S Ramesh[5], Srinath BS[6], Mohit Kumar Jolly[2], Tessy Thomas Maliekal[3], Jyothi S Prabhu[1]

Supplementary Figure S1: Expression level of *BIRC3*, *HIF1A*, *DICER* and *CDK19* after miR-18a inhibition as measured by q-PCR

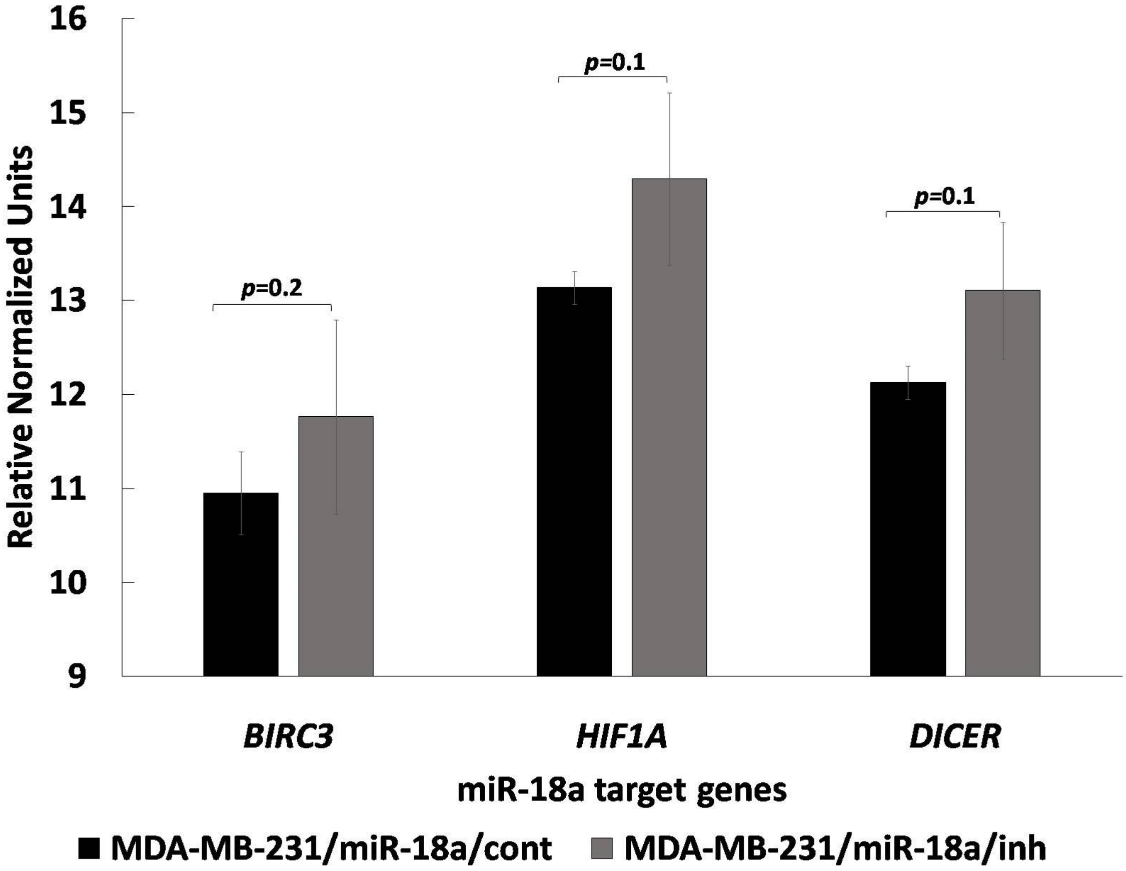

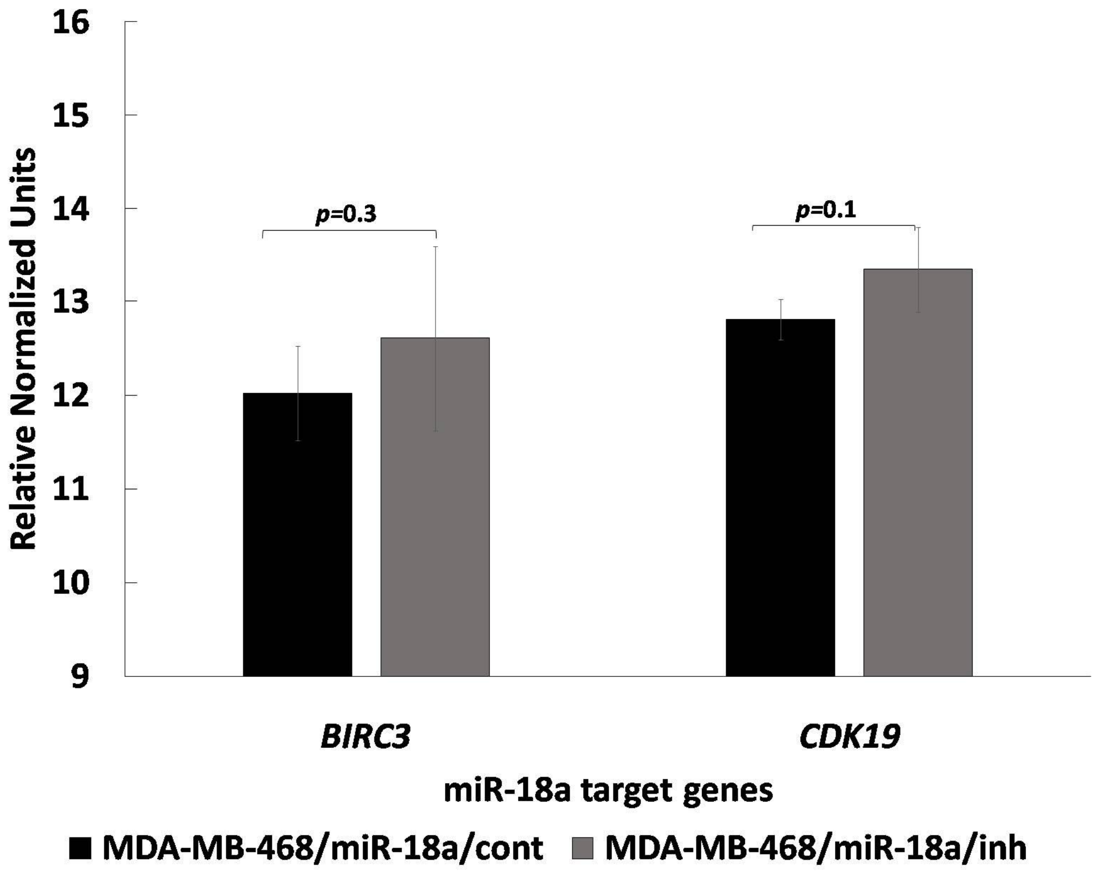
16
15
14
13
12
11
10
9
Relative Normalized Units
p=0.3
p=0.1
BIRC3
CDK19
miR-18a target genes
MDA-MB-468/miR-18a/cont
MDA-MB-468/miR-18a/inh

Supplementary Figure S2: Quantitative assessment of CD44 and CD24 expression in MDA-MB-453/miR-18a/cont and MDA-MB-453/miR-18a/inh as assessed by flow cytometry (cumulative result from 3 independent trials).

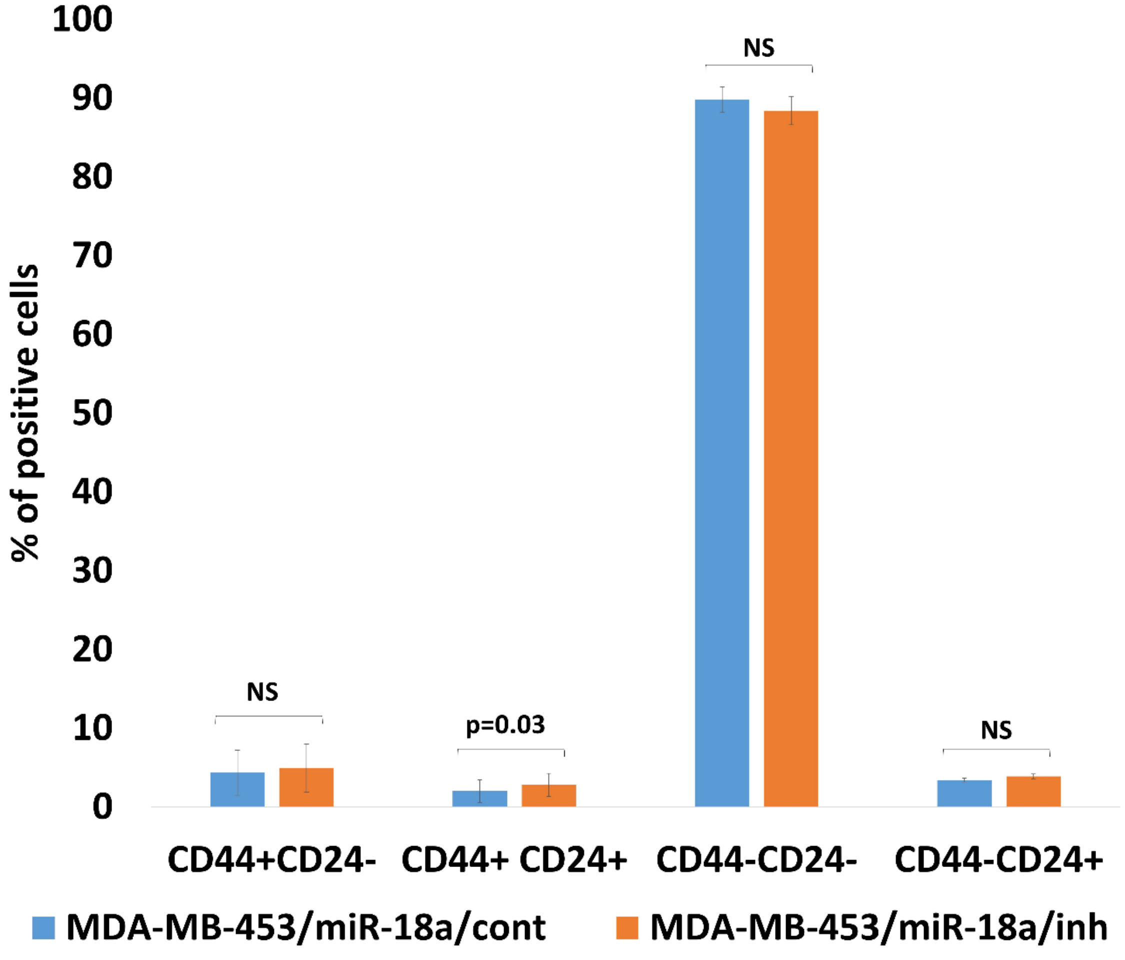

Supplementary Figure S3: Change in protein expression levels of MMP9 levels in MDA-MB-453/miR-18a/cont and MDA-MB-453/miR-18a/inh cells.

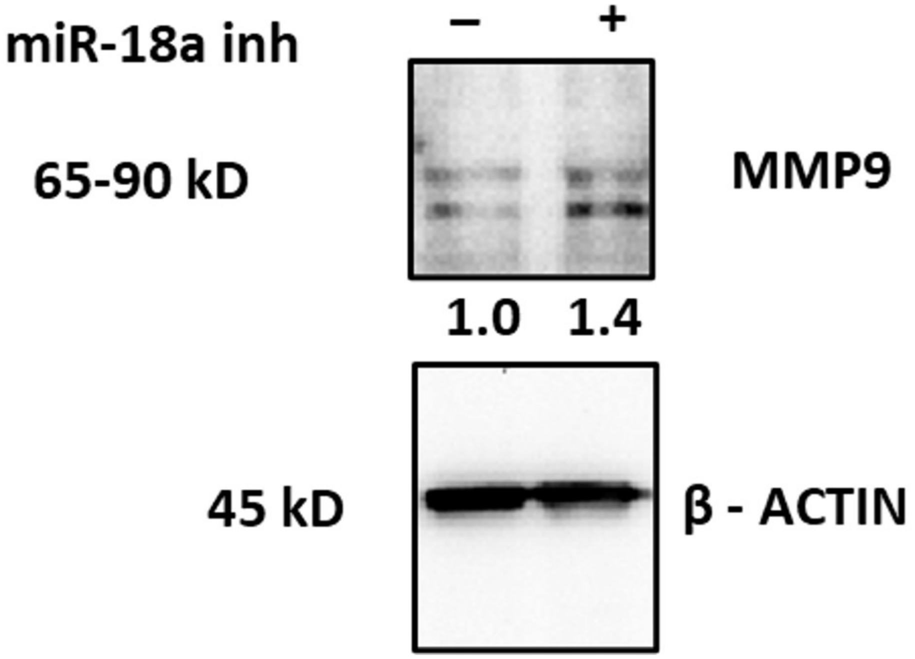

Supplementary Figure S4: DEGs associated with EMT and drug resistance in miR-18a/low, ER-negative tumours of METABRIC

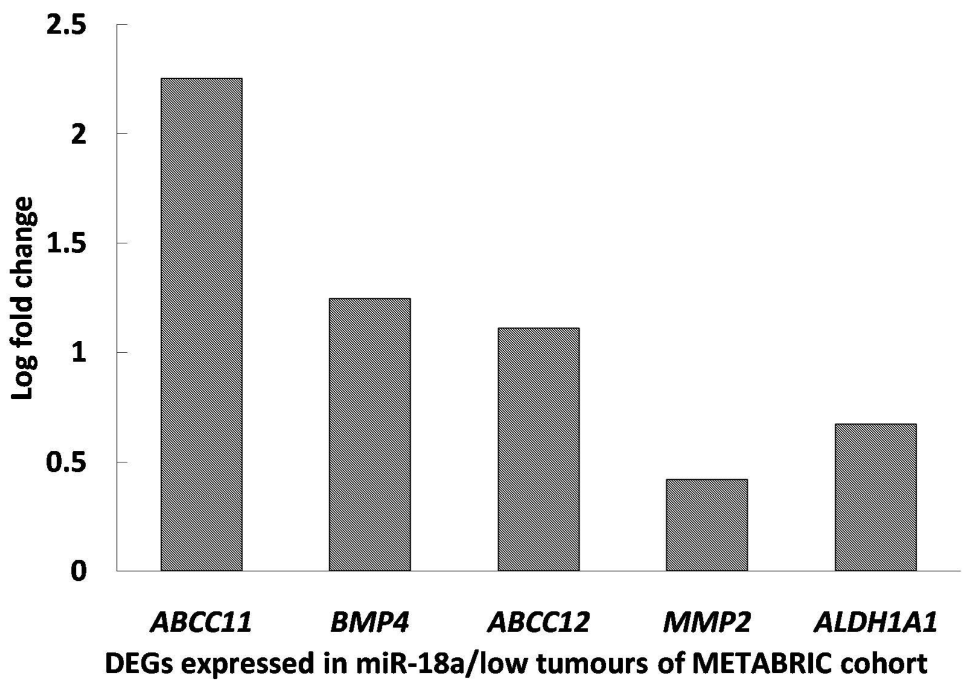

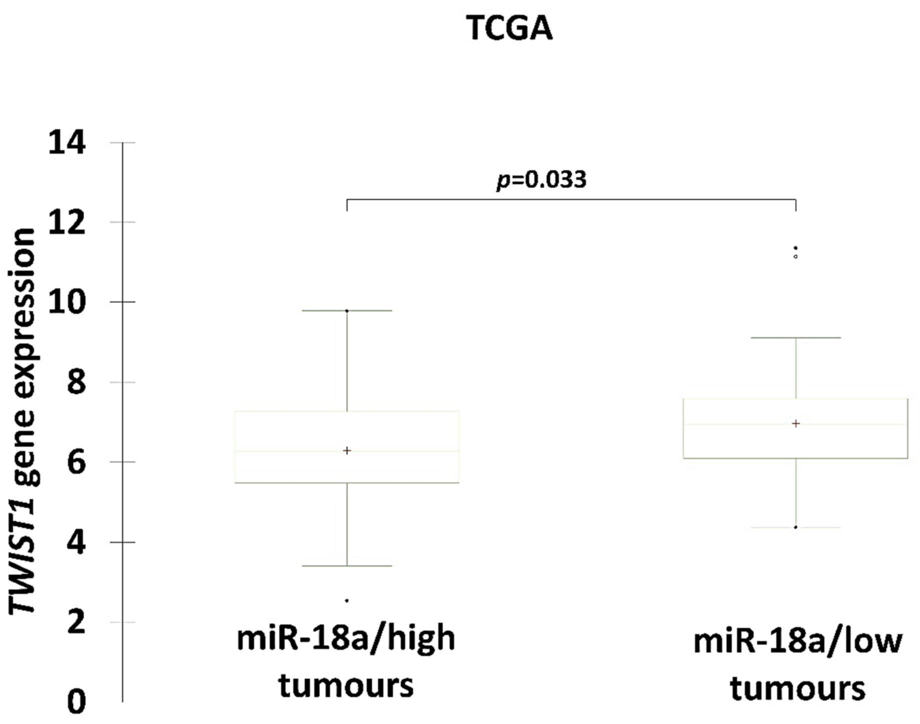

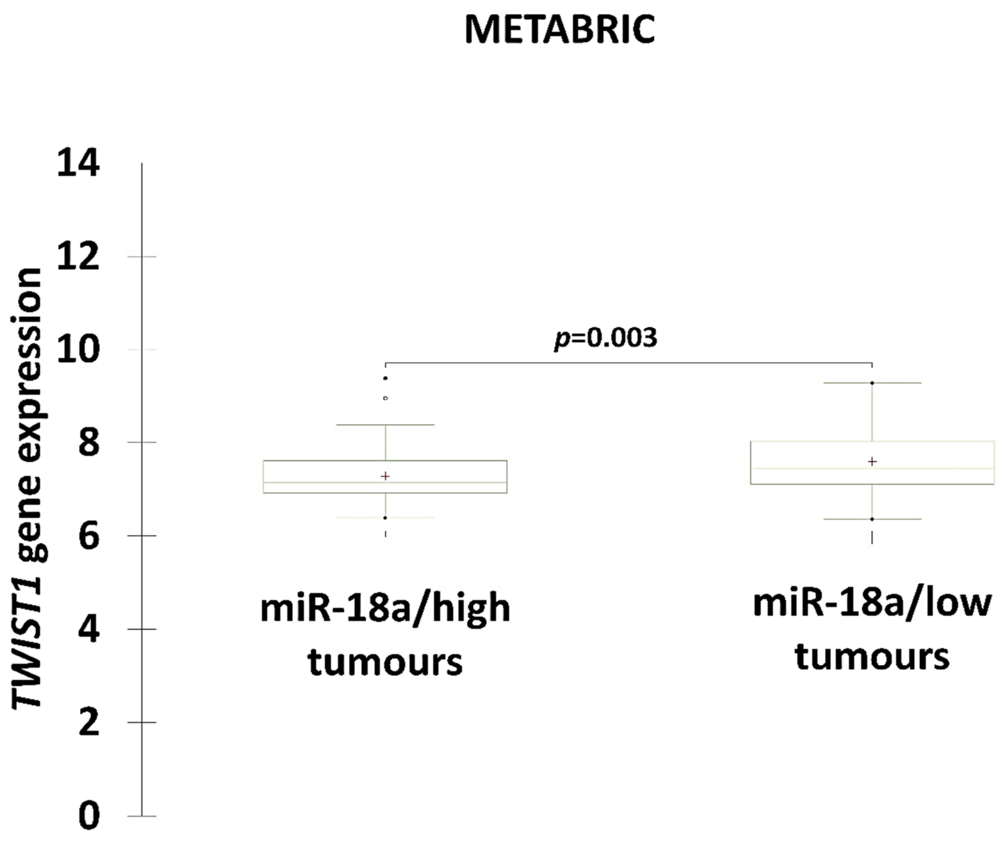
METABRIC
TWIST1 gene expression
14
12
10
8
6
4
2
0
p=0.003
miR-18a/high tumours
miR-18a/low tumours

Supplementary Figure S5a: The SI index in percentage calculated for MDA-MB-468/miR-18a/inh when compared to MDA-MB-468/miR-18a/cont (calculated with reference to MCF10A).

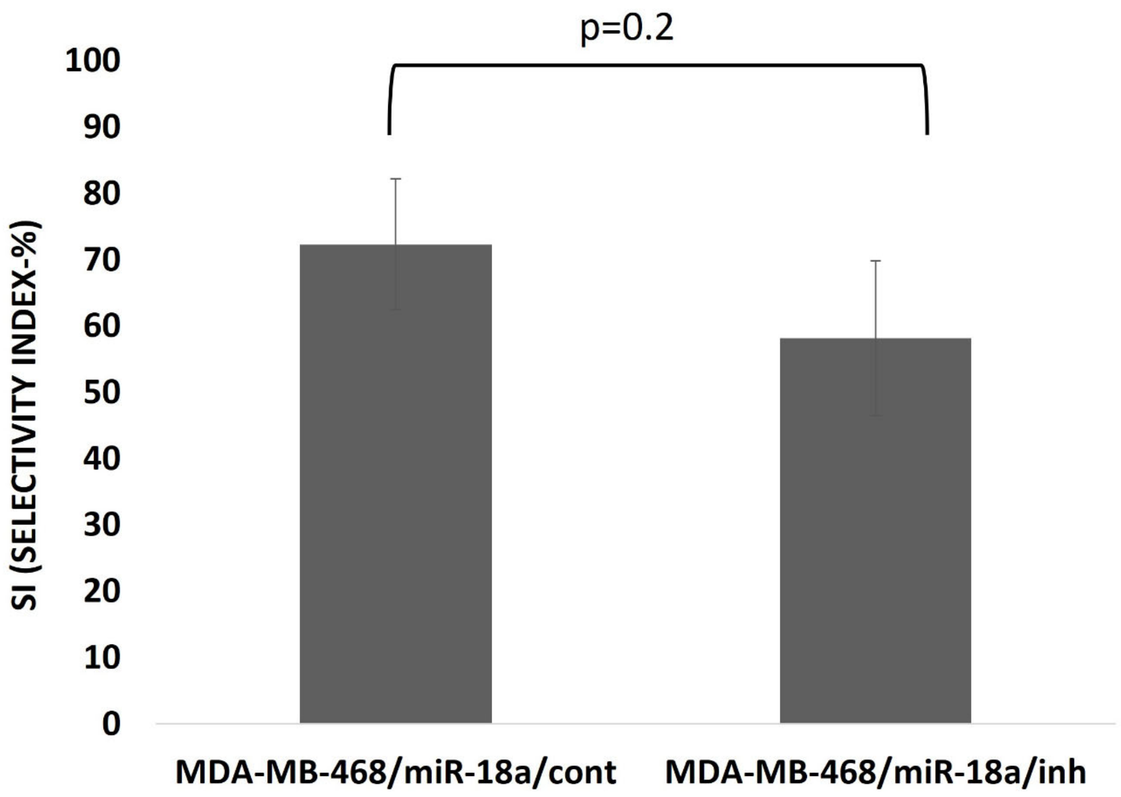

Supplementary Figure S5b: Expression level of stemness genes in ALDH1A1-DsRed2N1 cells vs DsRed2N1 cells.

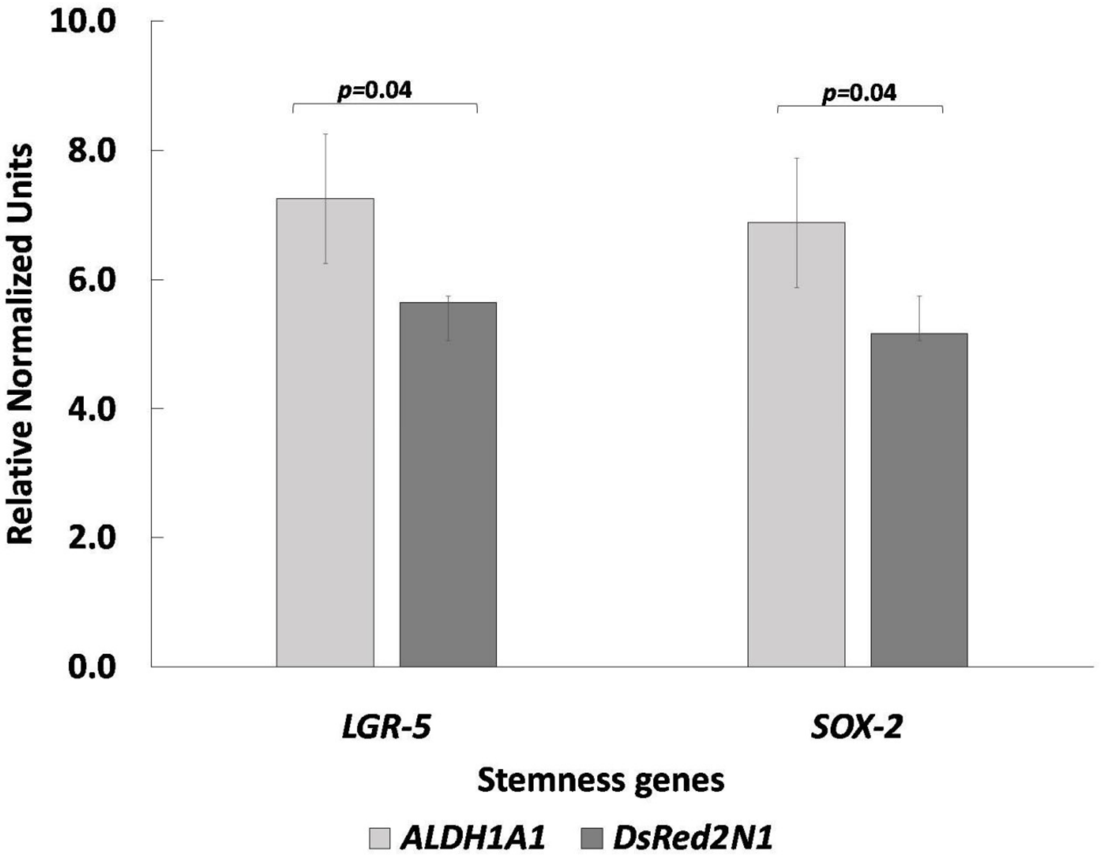

Supplementary Figure S6: Representative images of spheres obtained from MDA-MB-453/miR-18a/cont and MDA-MB-453/miR-18a/inh cells and table and graph depicting clonogenicity (stem cell frequency) calculated from extreme limiting dilution assay performed on MDA-MB-453/miR-18a/cont and MDA-MB-453/miR-18a/inh groups across the same initial seeding dose range of 1-1000 cells.

Cell number	MDA-MB-453 /miR/18a/control	MDA-MB-453 /miR/18a/inhibitor
1000	9	17
500	6	15
100	4	8
1	4	11
Stem cell frequency	1/929	1/233
p value = 9.69e-09		

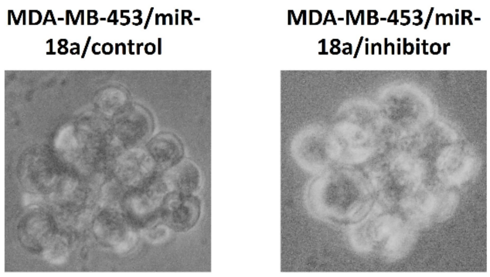

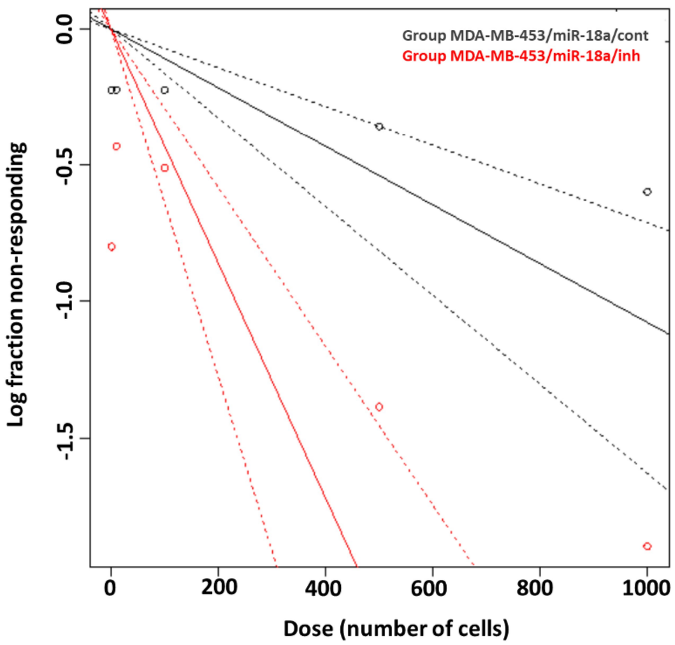

Supplementary Figure S7a: **CIBERSORT analysis depicting the proportions of M1 and M2 macrophages in miR-18a/high and miR-18a/low tumours of TCGA and METABRIC cohorts respectively, immune cell AI analysis depicting proportion of Type 2 T helper cells and Type 1 regulatory T cells in the METABRIC cohort.**

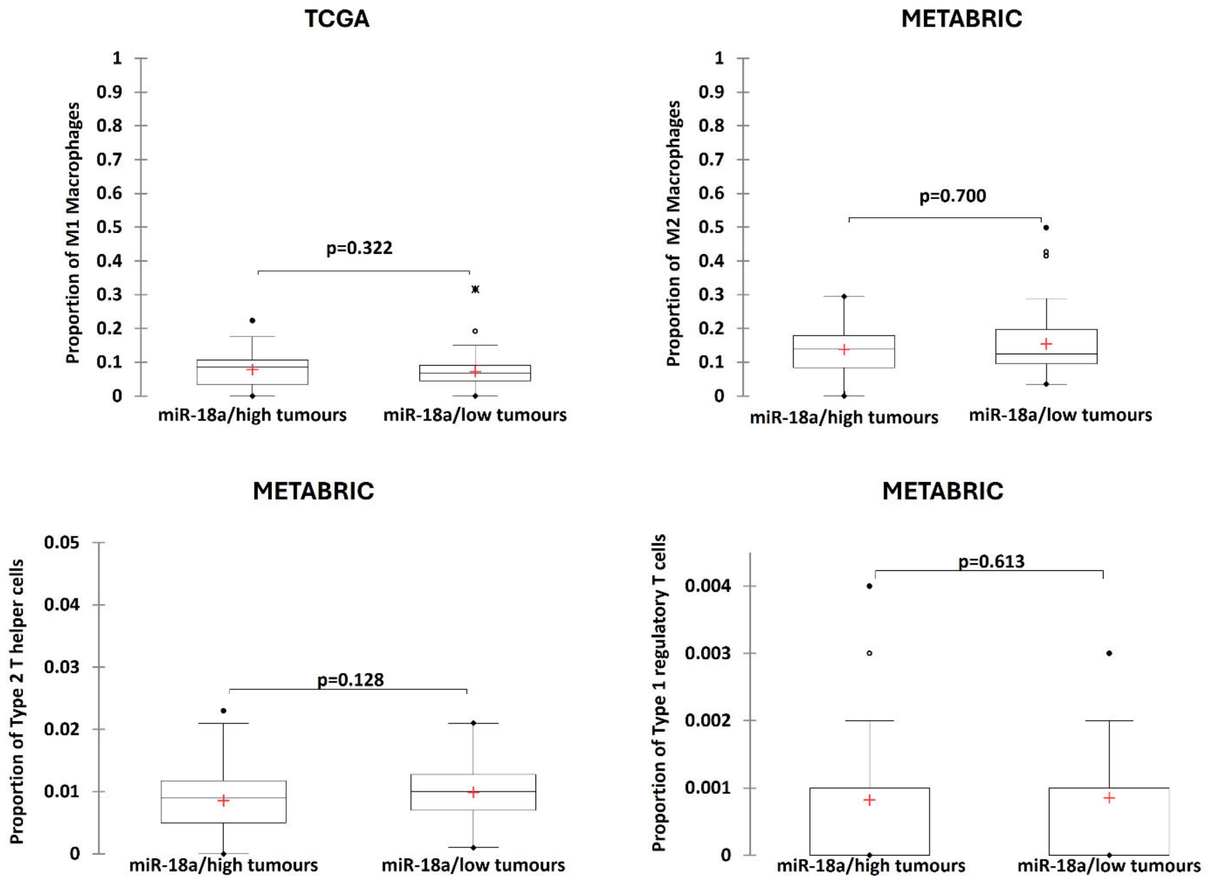

Supplementary Figure S7b: Change in protein expression levels of HIF-1α levels in MDA-MB-231/miR-18a/cont and MDA-MB-231/miR-18a/inh cells.

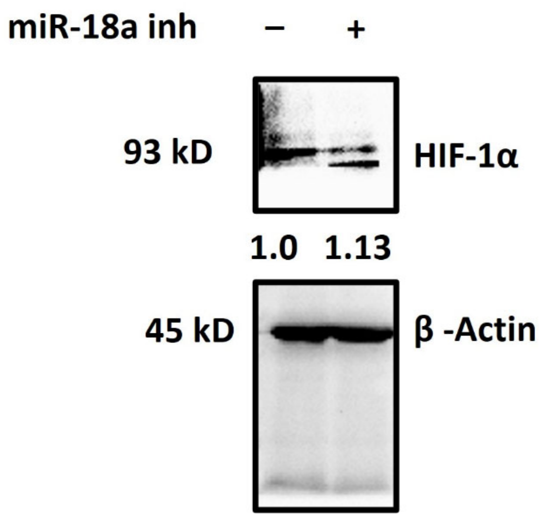

Supplementary Figure S7c: The heatmap representing the pattern of expression of filtered hypoxia genes in the miR-18a/low tumors of TCGA

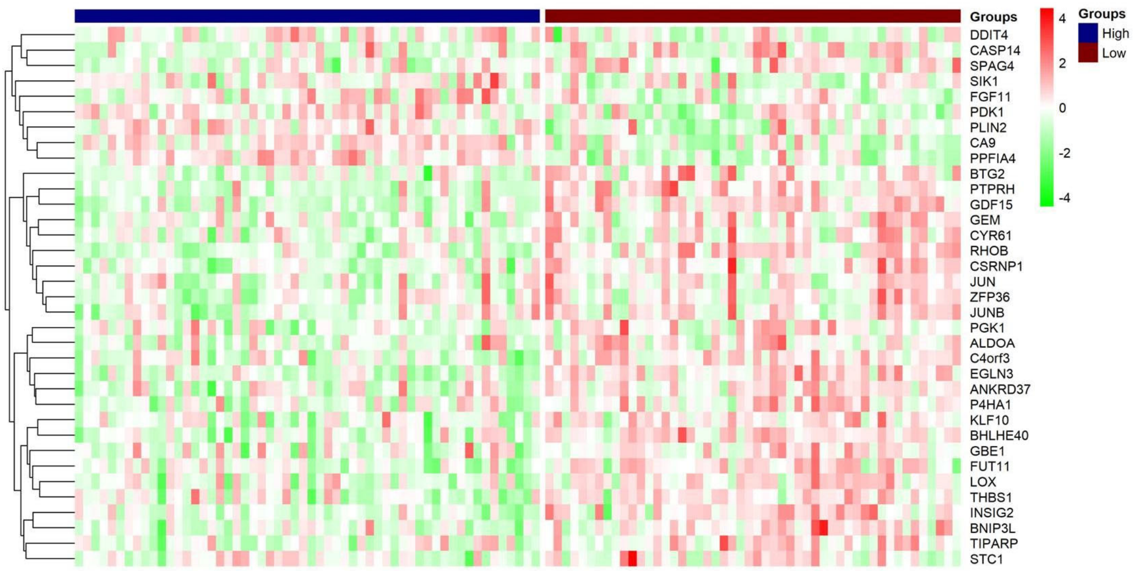

Supplementary Figure S7d: Representative H&E stained image of tumour from mice injected with miR-18a/antagomiR and and antagomiR negative control cells.

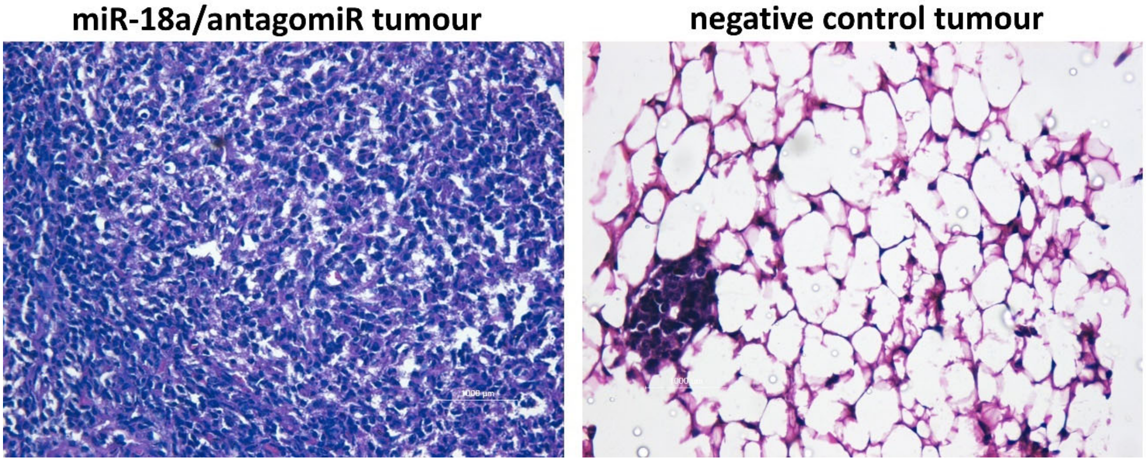

Supplementary Figure S7e: Change in protein expression levels of E-cadherin and Vimentin levels in MDA-MB-231/miR-18a/cont and MDA-MB-231/miR-18a/inh cells.

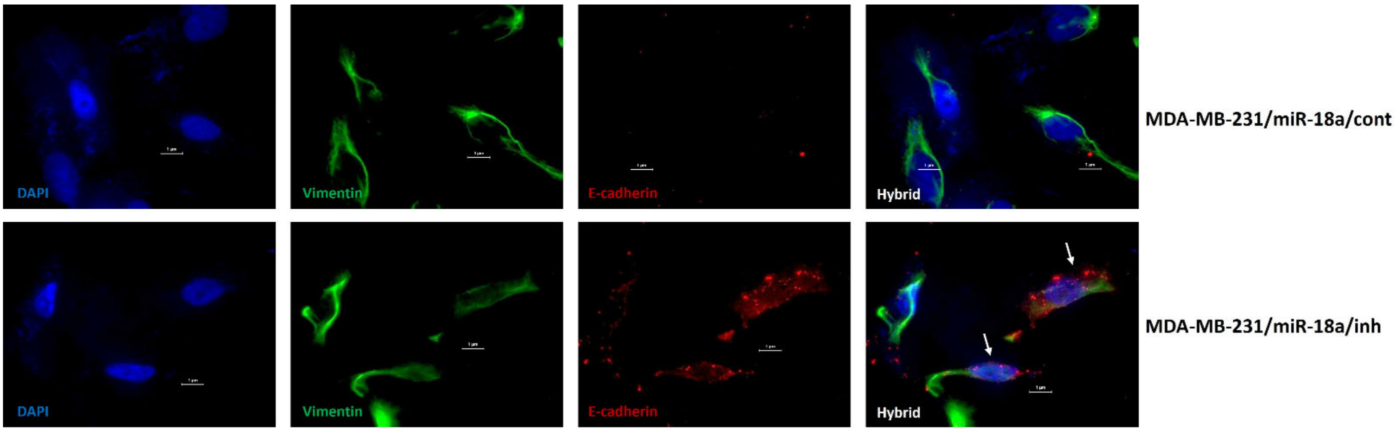

Supplementary Figure S8: Mutational spectrum analysis depicting higher PIK3CA mutation load in both TNBC and ER-HER2+ tumors of the miR-18a/low ER-negative tumours of METABRIC cohort.

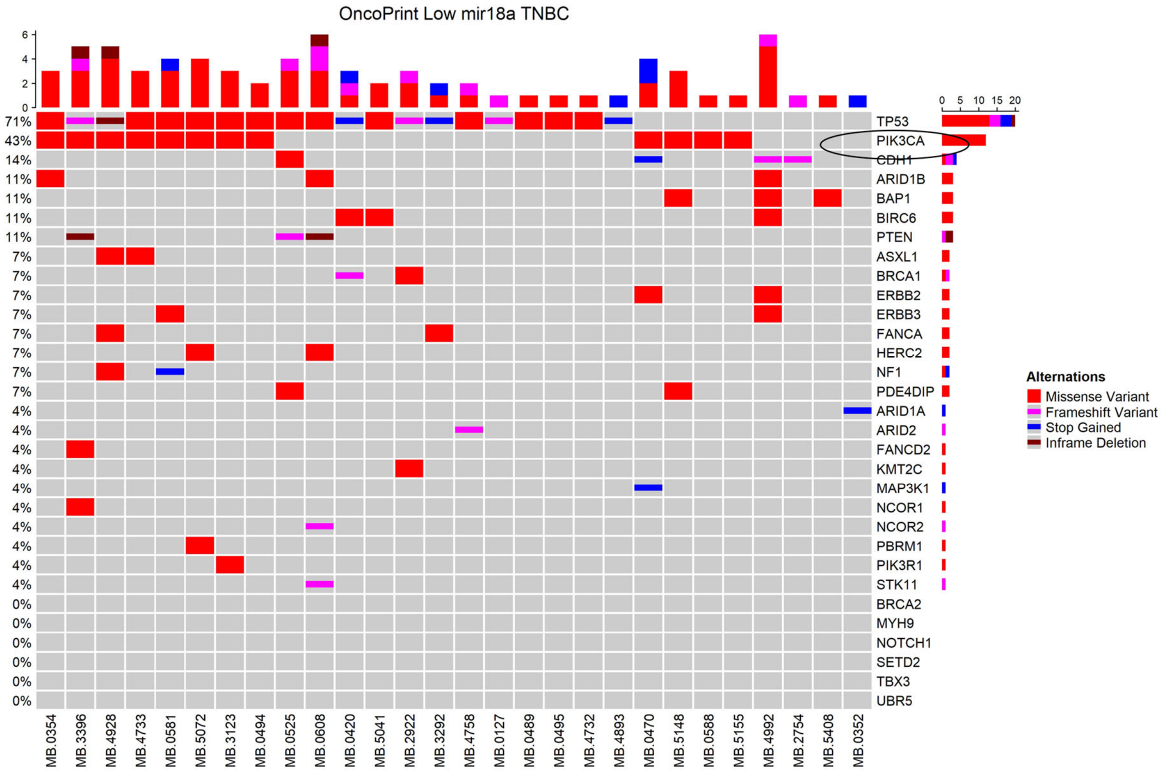

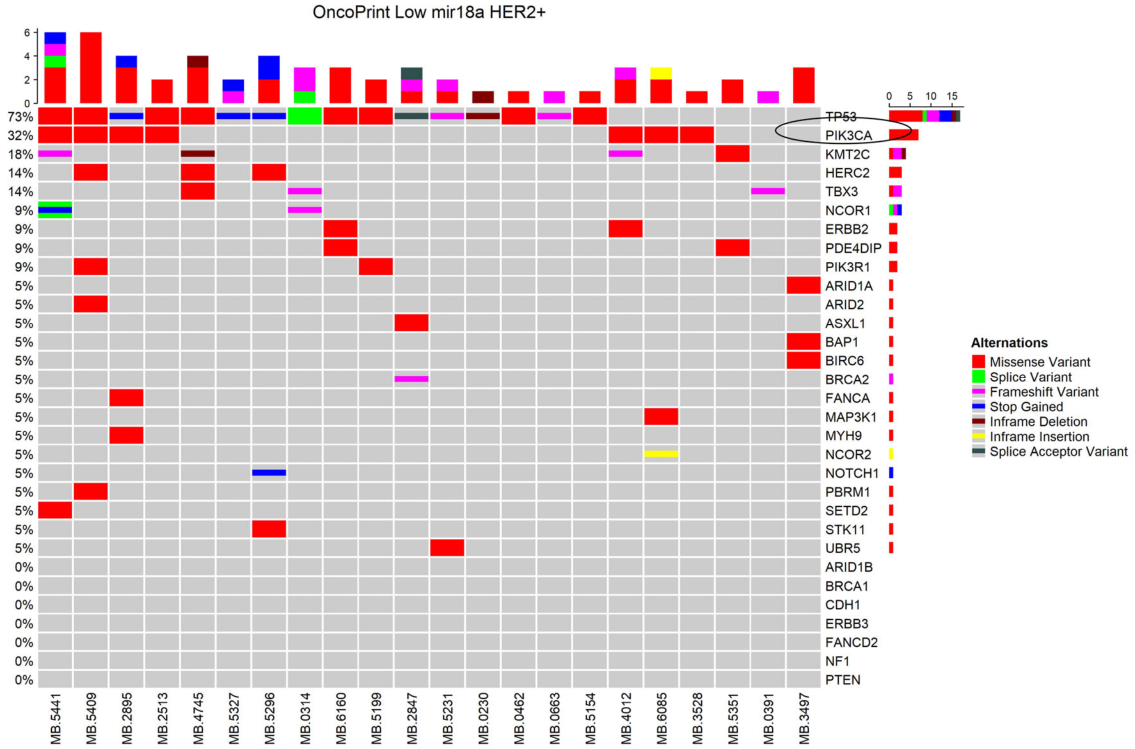

Supplementary Figure S9: Partial EMT score between the TNBC and ER-HER2+ tumors of the miR-18a/low ER-negative tumours of TCGA and METABRIC cohort.

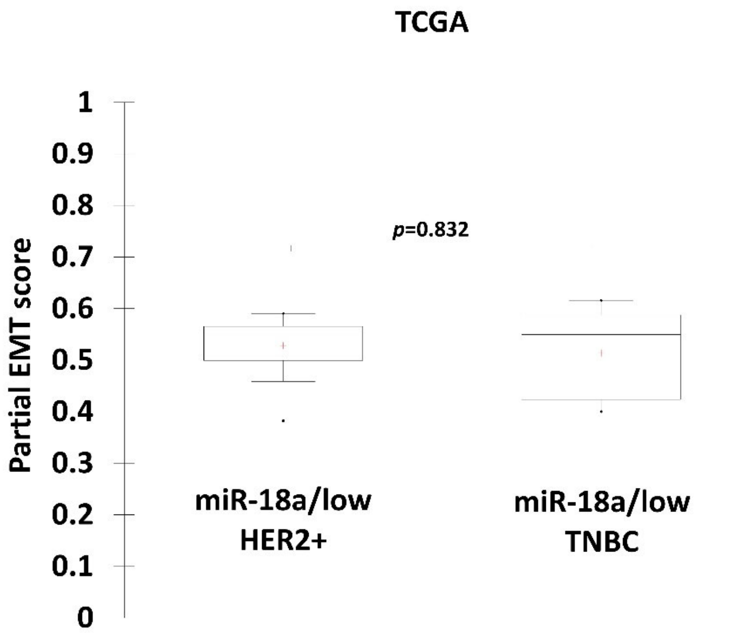

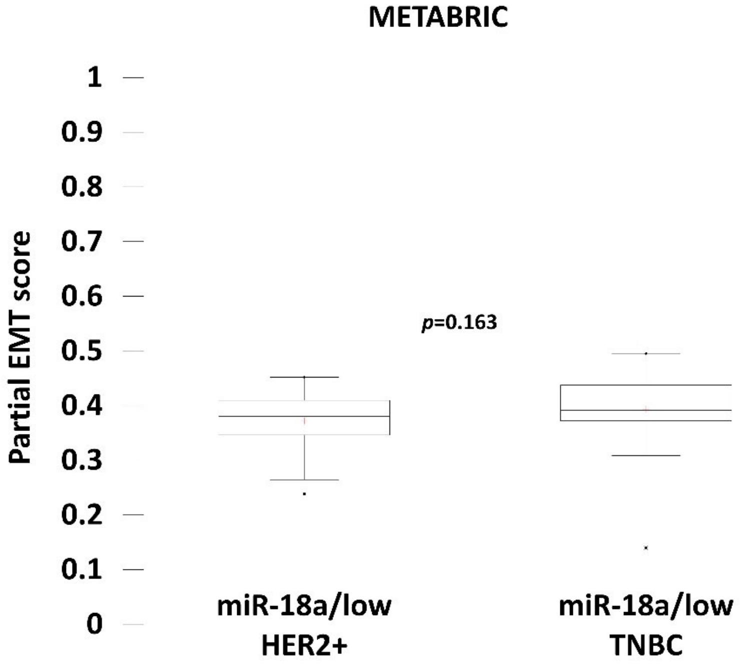

Partial EMT Score derived from - Puram SV, Tirosh I, Parikh AS, Patel AP, Yizhak K, Gillespie S, Rodman C, Luo CL, Mroz EA, Emerick KS, Deschler DG, Varvares MA, Mylvaganam R, Rozenblatt-Rosen O, Rocco JW, Faquin WC, Lin DT, Regev A, Bernstein BE. Single-Cell Transcriptomic Analysis of Primary and Metastatic Tumor Ecosystems in Head and Neck Cancer. Cell. 2017 Dec 14;171(7):1611-1624.e24. doi: 10.1016/j.cell.2017.10.044. Epub 2017 Nov 30. PMID: 29198524; PMCID: PMC5878932.

Supplementary Figure S10: 77 gene EMT Score between the TNBC and ER-HER2+ tumors of the miR-18a/low ER-negative tumours of the TCGA and METABRIC cohort.

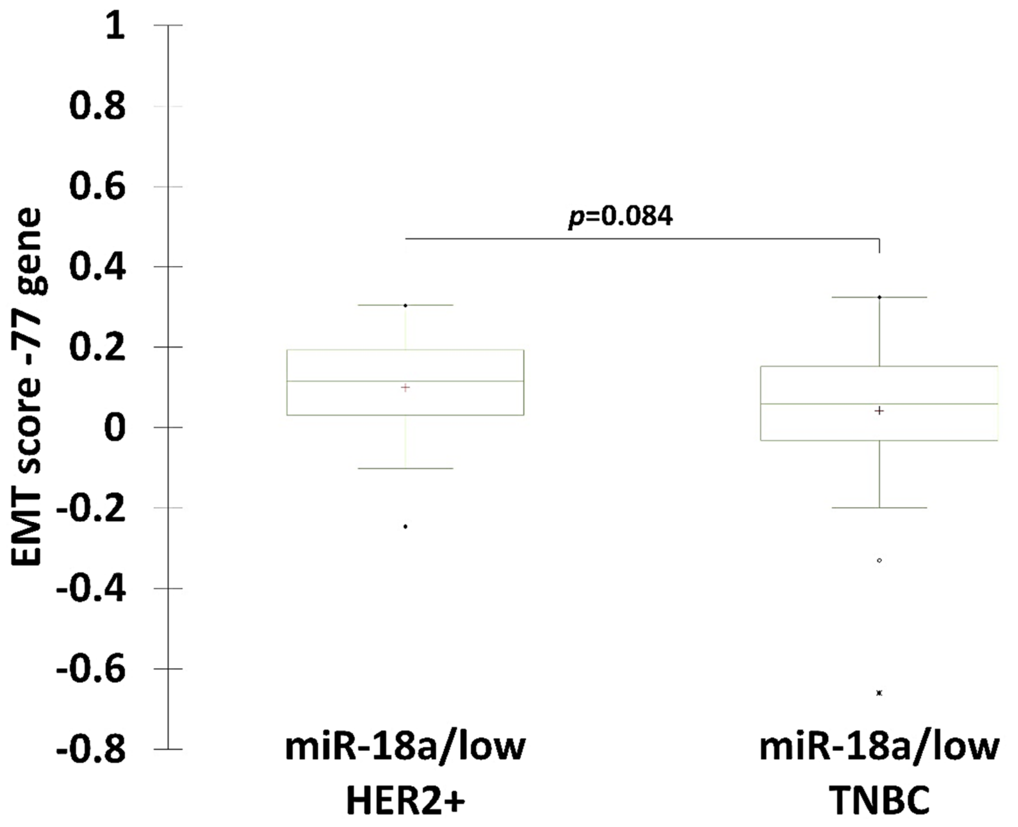

Supplementary Table S1 : List of antibodies and their dilutions

Sl. No	Antibody	Purpose	Product details	Dilution
1	TNFAIP3	Western Blot	Rabbit monoclonal EPR2663-ab92324 Abcam	1:1000
2	CD49f	Western Blot	Rabbit monoclonal EPR5578-ab124924 Abcam	1:5000
3	Cytokeratin-19	Western Blot	Rabbit monoclonal EP1580Y-ab52625 Abcam	1:10000
4	MMP9	Western Blot	Rabbit monoclonal- PA5-27191 Invitrogen	1:1000
5	RAC3	Western Blot	Rabbit monoclonal EPR6679(B)-ab124943 Abcam	1:5000
6	ALDH1	Western Blot	Mouse monoclonal- 611194 BD Biosciences	1:500
7	HIF1-α	Western Blot	Rabbit monoclonal EP1215Y-ab51608 Abcam	1:500
8	CD44	Flow cytometry	CD44 (156-3C11) Mouse mAb-3570 Cell signalling technology	1:100
9	CD24	Flow cytometry	CD24 (M1/69) Rat mAb PE Conjugate- 90378 Cell Signalling technology	1:660
10	E-cadherin	Western Blot /Immunofluorescence	Rabbit monoclonal EP700Y-ab40772 Abcam	1:5000/1:500
11	Vimentin	Immunofluorescence	Mouse clone V9- AM074-5M Bio-genex	1:25
12	Integrin β3	Immunohistochemistry	Rabbit monoclonal EPR2417Y-ab75872 Abcam	1:500

Supplementary Table S2. Clinico-pathological characteristics of 211 ER-negative patients used for analysis from TCGA dataset.

	All N (%) (n = 211)	miR-18a/low (n = 50)	miR-18a/high (n = 57)
**Age (y)**			
Mean	55	57	52
Median	55	58	51
**Tumor Size (cm)**			
T1	33 (22)	9 (26)	4 (10)
T2	100 (66)	19 (56)	34 (83)
T3	13 (9)	3 (9)	2 (5)
T4	5 (3)	3 (9)	1 (2)
**Stage**			
1	20 (14)	3 (9)	2 (5)
2	96 (65)	20 (63)	31 (76)
3	29 (20)	9 (28)	8 (19)
4	2 (1)		
**Lymph Node status**			
Positive	71 (39)	17 (49)	15 (30)
Negative	112 (61)	18 (51)	35 (70)
**Menopausal status**			
Pre	46 (24)	12 (26)	18 (36)
Post	142 (76)	35 (74)	32 (64)
**Her2 positivity**			
Negative	111 (62)	17 (40)	39 (76)
Positive	37 (21)	18 (43)	4 (8)
Equivocal	31 (17)	7 (17)	8 (16)

Supplementary Table S3. Clinico-pathological characteristics of 265 ER-negative patients used for analysis from METABRIC dataset.

	All N (%) (n = 265)	miR-18a/low (n = 54)	miR-18a/high (n = 62)
**Age (y)**			
Mean	55	59	53
Median	55	60	52
**Tumor Size (cm)**			
Mean	2.9	2.4	2.9
Median	2.5	2.2	2.5
**Stage**			
0	2 (1)	1 (2)	0 (0)
1	63 (27)	17 (34)	9 (15)
2	127 (55)	22 (44)	42 (71)
3	39 (17)	10 (20)	8 (14)
**Grade**			
I	2 (1)	1 (2)	0 (0)
II	33 (13)	11 (22)	3 (5)
III	223 (86)	38 (76)	58 (95)
**Lymph Node status**			
Positive	139 (52)	27 (50)	33 (53)
Negative	126 (48)	27 (50)	29 (47)
**Menopausal status**			
Pre	99 (37)	13 (24)	27 (44)
Post	166 (63)	41 (76)	35 (56)
**Her2 positivity**			
Negative	187 (71)	32 (59)	59 (95)
Positive	78 (29)	22 (41)	3 (5)

Supplementary Table S4: List of luminal and basal genes

Luminal genes	Basal genes
ESR1	*CK5/6*
GATA3	*Laminin*
TFF3	*CENPI*
FOXA1	*CENPK*
LIV-1	*CDC7*
KRT8	*KIF18A*
GRM4	*CCNE2*
GRM8	*STIL*
KRT18	*CDCA7*
PGR	*CKS2*
NMUR1	*MIA*
MUC1	*ANLN*
CX3CL1	*FABP7*
NCAM1	*KRT17*
XBP1	*KRT6b*
	DCS2

Supplementary Table S5. Clinico-pathological characteristics of 105 ER-negative patients used for analysis from our case series.

	**All N (%) (n= 105 patients)**
**Age (y)**	
Mean	54
Median	54
**Tumor Size (cm)**	
Mean	3.5
Median	3
**Stage**	
I	16 (15)
II	53 (50)
III	32 (30)
IV	4 (4)
**Grade**	
I	6 (6)
II	38 (39)
III	54 (55)
**Lymph Node status**	
Positive	54 (51)
Negative	49 (47)
Nx	2 (2)
**Menopausal status**	
Pre	28(27)
Post	77(73)
**Her2 positivity**	
Equivocal	4 (4)
Negative	72 (68)
Positive	29 (28)

Supplementary Table S6. Clinical characteristics of post NACT residual tumors: Clinico-pathological characteristics of (n=54) post NACT residual tumors and (n=43) tumors with adequate tissue available for estimation of integrin β3 and miR-18a

	n = 54 patients (%)	n = 43 patients (%)
**Age**		
Mean (Yrs)	49	49
Median (Yrs)	48	48
**Tumor Size**		
Mean (cm)	6	6
Median (cm)	6	6
**Stage**		
III	38 (70)	31 (72)
IV	14 (26)	10 (23)
Nx	2 (4)	2 (5)
**Menopausal status**		
Pre	24 (44)	19 (44)
Post	30 (56)	24 (56)
**Estrogen Receptor**		
Positive	30 (56)	22 (51)
Negative	24 (44)	21 (49)
**Progesterone Receptor**		
Positive	28 (52)	21 (49)
Negative	26 (48)	22 (51)
**HER2**		
Positive	18 (33)	13 (30)
Negative	29 (54)	25 (58)
Equivocal	7 (13)	5 (12)

Supplementary Table S7: List of primer sequences used in q-RT-PCR analysis

Sl No.	Gene	PRIMER SEQUENCE
1	*ACTB*	5'-TTCCTGGGCATGGAGTC-3'<br>3'-CAGGTCTTTGCGGATGTC-5'
2	*ANLN*	5'-ACAGCCACTTTCAGAAGCAAG-3'<br>3'-CGATGGTTTTGTACAAGATTTCTC-5'
3	*BCL2*	5'-TACCTGAACCGGCACCTG-3'<br>3'-GCCGTACAGTTCCACAAAGG-5'
4	*BIRC3*	5'-CCATGGGTTCAACATGCCAAGTGGT-3'<br>3'-GGGTAACTGGCTTGAACTTGACGG-5'
5	*BMPR1B*	5'-ATTCCCAAACCGGTGGAGCAGT-3'<br>3'-TTGATGCAGGATTGTGAGCCCAGC-5'
6	*CDK19*	5'-TACCTCCATGCAAATTGGGTGCT-3'<br>3'-R-TTTGACTCTCCCCCTCTCAGGA-5'
7	*DICER*	5'-TTAACCTTTTGGTGTTTGATGAGTGT-3'<br>3'-GCGAGGACATGATGGACAATT-5'
8	*HIF1A*	5'-TGCTTACACACAGAAATGGCCT-3'<br>3'-TAGTTAGGGTACACTTCATTCTGAG-5'
9	*ITGB3*	5'-CCCACCAGAGGCCCTCGAAA-3'<br>3'-AAGCGGGTCACCTGGTCAGT-5'
10	*LGR5*	5'-GACCATTGCCTACACCAAGC-3'<br>3'-GAGCAACAGGGCAATGTGTT-5'
11	*PGR*	5'-TTATAATTCGAGGCGGTTAGTGTTT-3'<br>3'-TCGAACTTCTACTAACTCCGTACTACGA-5'
12	*PUM1*	5'-CCGGAGATTGCTGGACATATAA-3'<br>3'-TGGCACGCTCCAGTTTC-5'
13	*RPLP0*	5'-GGCTGTGGTGCTGATGGGCAAGAA-3'<br>3'-TTCCCCCGGATATGAGGCAGCAGT-5'
14	*SALL4*	5'-GGCCAATAGTCAAGCCGAAA-3'<br>3'-TCCGACCTTCCATCTCAGTG-5'
15	*ZEB2*	5'-TGCACAGAGTGTGGCAAGGC-3'<br>3'-TGGGCACTCGTAAGGTTTTTCACC-5'

Details on literature survey:

The literature survey as part of the manuscript was conducted using Pubmed search and Google scholar search. The key words employed for the search was ‘miR-18a ER-Negative breast cancer’, ‘miR-18a cancer’, ‘miRNA targeting’.

The time period for literature survey was 2009-2022.

Western blot - Densitometric analysis and raw blot images

Fig 1B
TNFAIP3

	Control	miR-18a inhibitor
TRIAL 1	100	103.1
TRIAL 2	100	157.5
TRIAL 3	100	155.8959
TRIAL 4	100	164.5387
TRIAL 5	100	147.2625
AV	100	145.6594
STD ERROR	0	10.98928
P VALUE		0.000236

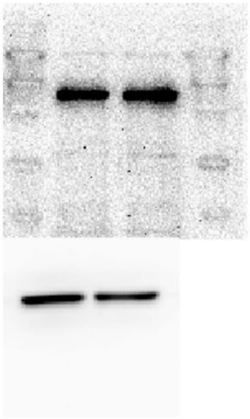

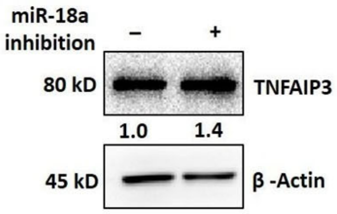

Fig 1B
CD49f

	Negative control	miR-18a inhibitor
TRIAL 1	100	270
TRIAL 2	100	258
TRIAL 3	100	158
AV	100	208
STD ERROR	0	40.82483
P VALUE	0.000668	

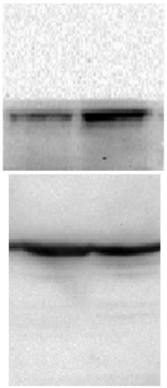

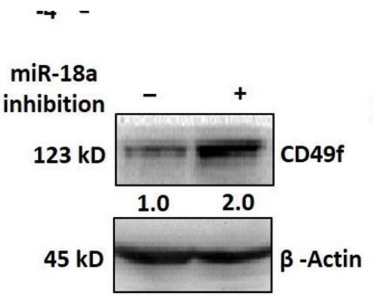

Fig 1B
Cytokeratin 19

	Negative control	miR-18a inhibitor
TRIAL 1	100	181
TRIAL 2	100	149
TRIAL 3	100	125
TRIAL 4	100	151.6667
AV	100	151.6667
STD ERROR	0	16.2207
P VALUE	0.016684	

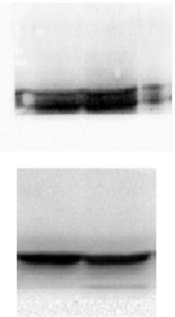

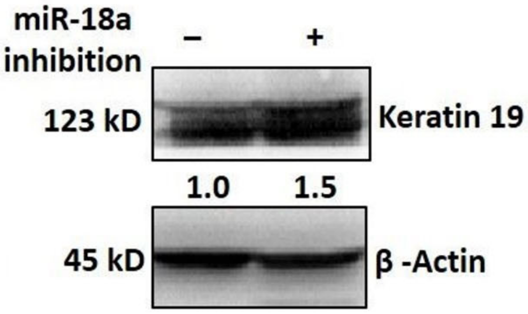

Fig 2C
E-cadherin

	Negative control	miR-18a inhibitor
TRIAL 1	100	86
TRIAL 2	100	73.2
TRIAL 3	100	94.5
AV	100	84.56666667
STD ERROR	0	6.190404761
P VALUE	0.03363	

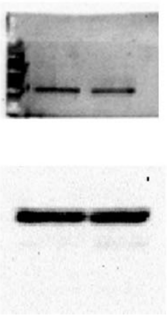

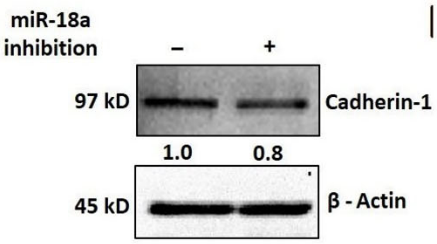

Fig 2C MMP9

	Negative control	miR-18a inhibitor
TRIAL 1	100	137.2
TRIAL 2	100	171.8
TRIAL 3	100	157.8
AV	100	155.6
STD ERROR	0	10.04855
P VALUE	0.002607	

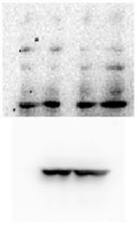

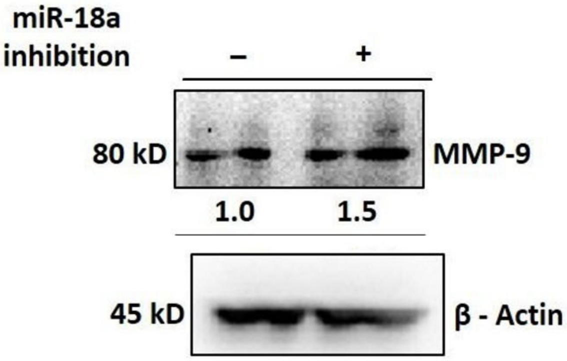

Fig 2C RAC3

	Negative control	miR-18a inhibitor
TRIAL 1	100	147
TRIAL 2	100	150
TRIAL 3	100	131.5
AV	100	142.8333
STD ERROR	0	5.732461
P VALUE	0.000857	

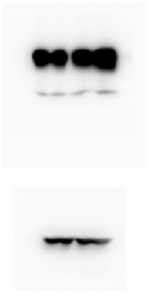

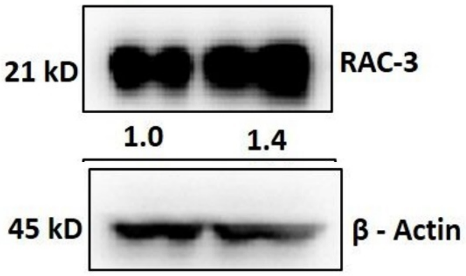

Fig 3H ALDH1A1

	Negative control	miR-18a inhibitor
TRIAL 1	100	115
TRIAL 2	100	191
TRIAL 3	100	144
AV	100	150
STD ERROR	0	22.14347
P VALUE	0.043436135	

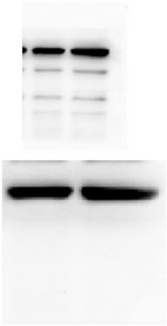

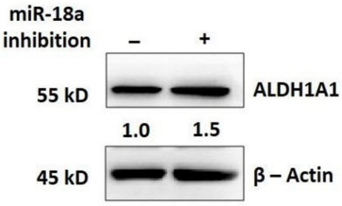

Fig 5C HIF1A

	Negative control	miR-18a inhibitor
TRIAL 1	100	331.2888
TRIAL 2	100	179.2266
TRIAL 3	100	147.3008
AV	100	219.2721
STD ERROR	0	56.76155
P VALUE	0.051752	

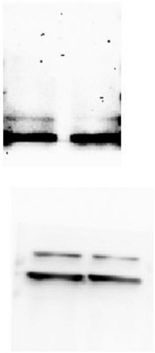

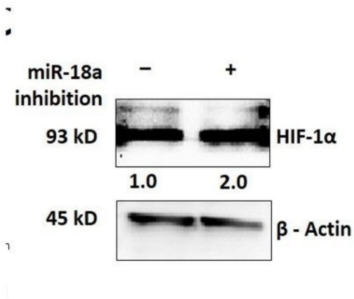

Fig S3 MMP9

	Negative control	miR-18a inhibitor
TRIAL 1	100	144.5632
TRIAL 2	100	191.7947
TRIAL 3	100	110.622
AV	100	148.9933
STD ERROR	0	23.53699
P VALUE	0.052919	

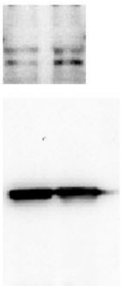

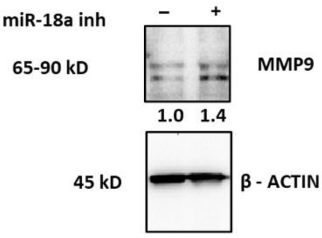

Supplement: Supplementary file 1 [file cells-13-00821-s001.zip › cells-2915996-supplementary.pdf]
